# Assessing the suitability of mitochondrial and nuclear DNA genetic markers for molecular systematics and species identification of helminths

**DOI:** 10.1186/s13071-021-04737-y

**Published:** 2021-05-01

**Authors:** Abigail Hui En Chan, Kittipong Chaisiri, Sompob Saralamba, Serge Morand, Urusa Thaenkham

**Affiliations:** 1 Department of Helminthology, Faculty of Tropical Medicine, Mahidol University, Bangkok, Thailand; 2 Mathematical and Economic Modelling (MAEMOD), Mahidol Oxford Tropical Medicine Research Unit, Faculty of Tropical Medicine, Mahidol University, Bangkok, Thailand; 3 CNRS ISEM-CIRAD ASTRE, Faculty of Veterinary Medicine, Kasetsart University, Bangkok, Thailand

**Keywords:** Genetic marker, Molecular systematics, Molecular identification, Helminth, K-means

## Abstract

**Background:**

Genetic markers are employed widely in molecular studies, and their utility depends on the degree of sequence variation, which dictates the type of application for which they are suited. Consequently, the suitability of a genetic marker for any specific application is complicated by its properties and usage across studies. To provide a yardstick for future users, in this study we assess the suitability of genetic markers for molecular systematics and species identification in helminths and provide an estimate of the cut-off genetic distances per taxonomic level.

**Methods:**

We assessed four classes of genetic markers, namely nuclear ribosomal internal transcribed spacers, nuclear rRNA, mitochondrial rRNA and mitochondrial protein-coding genes, based on certain properties that are important for species identification and molecular systematics. For molecular identification, these properties are inter-species sequence variation; length of reference sequences; easy alignment of sequences; and easy to design universal primers. For molecular systematics, the properties are: average genetic distance from order/suborder to species level; the number of monophyletic clades at the order/suborder level; length of reference sequences; easy alignment of sequences; easy to design universal primers; and absence of nucleotide substitution saturation. Estimation of the cut-off genetic distances was performed using the ‘K-means’ clustering algorithm.

**Results:**

The nuclear rRNA genes exhibited the lowest sequence variation, whereas the mitochondrial genes exhibited relatively higher variation across the three groups of helminths. Also, the nuclear and mitochondrial rRNA genes were the best possible genetic markers for helminth molecular systematics, whereas the mitochondrial protein-coding and rRNA genes were suitable for molecular identification. We also revealed that a general gauge of genetic distances might not be adequate, using evidence from the wide range of genetic distances among nematodes.

**Conclusion:**

This study assessed the suitability of DNA genetic markers for application in molecular systematics and molecular identification of helminths. We provide a novel way of analyzing genetic distances to generate suitable cut-off values for each taxonomic level using the ‘K-means’ clustering algorithm. The estimated cut-off genetic distance values, together with the summary of the utility and limitations of each class of genetic markers, are useful information that can benefit researchers conducting molecular studies on helminths.
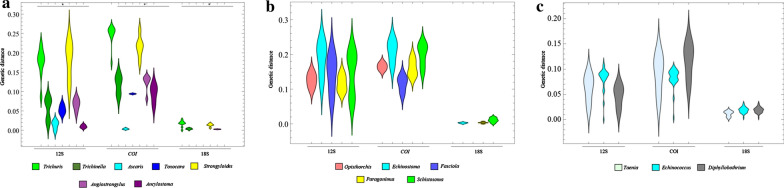

**Supplementary Information:**

The online version contains supplementary material available at 10.1186/s13071-021-04737-y.

## Background

Advances in molecular biology have accelerated the use of various molecular techniques and genetic markers in the fields of molecular systematics and taxonomy. Molecular genetic markers are DNA segments of the genome that can provide molecular information enabling the differentiation of taxa [1, 2]. The use of DNA sequences as genetic markers has proven successful not only for species identification and the discovery of new species but also for elucidating relationships between groups of organisms in systematics studies [3, 4].

Genetic markers can be designed from different DNA regions from either the nuclear or mitochondrial genomes. The utility and resolution of each genetic marker depend highly on the degree of sequence variation of the marker [5]. Compared to nuclear DNA (nDNA), mitochondrial DNA (mtDNA) evolves faster, thereby producing a higher degree of sequence variation, which makes it a potentially useful source of genetic markers to resolve lower taxonomic levels for organisms [6–9]. Within mtDNA, examples of genetic markers include the protein-coding genes of the cytochrome *c* oxidase subunit I (*COI*) and the NADH dehydrogenase subunit 1 (*NAD*1) genes and the 12S and 16S ribosomal RNA (rRNA) genes. Conversely, nDNA, particularly the nuclear rRNA genes, is more conserved than mtDNA. The highly conserved sequences make the nuclear rRNA genes a potentially helpful source of genetic markers for resolving higher taxonomic levels for organisms [1, 6, 10]. Within nDNA, the internal transcribed spacer (ITS) regions possess a higher degree of sequence variation than the nuclear rRNA genes because of a faster nucleotide substitution rate [6, 8, 10, 11]. Although multiple types of genetic markers are suitable for molecular systematics and identification purposes, the varying properties of the genetic markers complicate the choice for their respective applications.

Many studies have utilized genetic markers for molecular systematics and identification studies within the three groups of helminths (nematodes, cestodes, trematodes). Although the three groups of helminths are collectively grouped together, due in part to their parasitic lifestyle, nematodes and platyhelminths (cestodes and trematodes) are phylogenetically far apart from each other [12]. In molecular systematics, the nuclear 18S rRNA gene has been used to provide a phylogenetic framework for classifying and understanding relationships within helminths [13–15]. The successful use of the 18S rRNA gene for classification has prompted researchers to expand the range of taxa studied to increase the number of species sampled [16–19]. A combination of the 18S and 28S rRNA genes has been studied as a strategy to increase the resolution in cestode and trematode systematics [15, 20–22]. Even more recently, de León et al. provided a comprehensive and updated phylogeny of Digenea with the nuclear rRNA genes [23]. For the purposes of molecular identification, the nuclear ITS regions have been utilized successfully for species differentiation because of their high degree of sequence variation. Numerous studies have demonstrated the applicability of species-specific ITS primers to identify helminths for diagnostic purposes [10, 24, 25]. MtDNA genes have also been used to discriminate between species and populations [26–28]. For example, mtDNA genes have been used as genetic markers for successful interspecific discrimination among several helminth species, including among *Taenia* spp. for cestodes, *Echinostoma* and *Schistosoma* spp. for trematodes and *Trichuris* spp. for nematodes [29–31]. In addition, many molecular-based studies have also utilized more than one DNA genetic marker to assess phylogenetic relationships within the organism of interest. The benefit of using more than one marker is that congruence between the phylogenies obtained can be compared [32, 33].

Despite the many successes reported in using genetic markers in molecular studies of helminths for systematics and identification purposes, there is a caveat. Estimates to determine what constitutes ‘sufficient’ genetic variation among taxa and across taxonomic levels using genetic distances varies, depending on the genetic marker used and the taxa studied. Researchers have used genetic distance widely, at both the inter-and intra-species level, as a gauge for deciding whether specimens are conspecific. In general, a genetic difference of approximately 10% among closely related species using mitochondrial protein-coding genes is the basis for comparison to determine if the specimens are conspecific [8]. Species limits are also typically established following morphologically based information, and the DNA information is then fitted into the pre-defined classification. Various models have been developed to estimate species and taxonomic boundaries for different groups of organisms. For example, Pons et al*.* used a likelihood ratio test to assess the fit of phylogenetic tree branch lengths to define putative species and set species boundaries for insects [34]. The Poisson Tree Processes model, proposed by Zhang et al. with arthropod and lizard datasets, used speciation rates to delimit species through the number of substitutions [35]. Another method, developed using a multispecies coalescent model, was developed for simultaneous Bayesian inference of species delimitation and phylogeny [36]. Given that each genetic marker has different properties and nucleotide substitution rates, and that different groups of organisms have different evolutionary rates, we propose here potential estimates of genetic distances to aid in the classification and identification of helminths.

The aim of this study was to assess the suitability of nuclear and mitochondrial genetic markers for molecular systematics and identification purposes. The aim was achieved by comparing the properties of four classes of genetic markers (nuclear rRNA genes, nuclear ribosomal ITS regions, mitochondrial protein-coding genes, and mitochondrial rRNA genes) across taxonomic hierarchy levels to ascertain their suitability for molecular systematics and species identification. Secondly, we aimed to estimate a cut-off for each genetic marker using the ‘K-means’ clustering method with genetic distances. The ‘K-means’ clustering approach has been extensively used in various applications, including DNA sequences for phylogenetic analysis and identifying invasive weed species [37–39].

Our ultimate goal was to provide a guide for researchers studying future applications of genetic markers, in terms of molecular systematics and species identification, for helminths, through our assessment of genetic markers and estimation of cut-off genetic distance values.

## Methods

### Selection of representative taxa and sequences for each genetic marker

Four classes of genetic markers comprising both mtDNA and nDNA were selected for study: mitochondrial protein-coding genes, mitochondrial rRNA genes, nuclear rRNA genes and nuclear ribosomal ITS regions. *COI*, cytochrome *c* oxidase subunit II (*COII*), cytochrome* B* (*cytb*) and *NAD*1 genes represent the mitochondrial protein-coding genes; 12S and 16S rRNA genes represent the mitochondrial rRNA genes; 18S and 28S rRNA genes represent the nuclear rRNA genes; and the ITS1 and ITS2 regions represent the nuclear ribosomal ITS regions.

We obtained full-length sequences of mtDNA genes from the complete mitochondrial genomes of each helminth species contained in the National Center for Biotechnology Information (NCBI) database (www.ncbi.nlm.nih.gov). In all, we used 142 sequences of the mtDNA genes from helminths of medical importance to humans and animals for our analyses: 64 sequences from nematodes, 28 from trematodes and 45 from cestodes. We also obtained close to full-length sequences from the NCBI database for the nuclear rRNA genes and nuclear ribosomal ITS regions. The number of sequences used are as follows: 18S rRNA—47 from nematodes, 33 from trematodes, 44 from cestodes; 28S rRNA—27 from nematodes, 42 from trematodes, 16 from cestodes; ITS1—32 from nematodes, 30 from trematodes, 14 from cestodes; ITS2—29 from nematodes, 29 from trematodes, 12 from cestodes. As best as possible, we selected sequences from the nuclear DNA genetic markers from the same species from which we obtained from the mtDNA genetic markers. When no sequence was available for the same species, we selected congeneric sequences. The sequences for each group of helminths and each genetic marker used in this study are listed in Additional file 1: Table S1.

### Assessment of the suitability of each genetic marker for molecular systematics and molecular identification purposes

At present, there is no fixed set of criteria to determine which genetic marker is the most ideal for each application. Thus, we have generated a list of properties important for choosing suitable genetic markers for molecular systematics and molecular identification purposes. Ideally, the genetic marker should have an optimal evolution rate to provide sufficient informative sites for phylogenetic analysis and molecular identification. The marker should also show high interspecific variation between closely related species, which can be assessed through genetic distances to ascertain whether the marker has ‘sufficient’ sequence variation between organisms [1, 9, 10, 40, 41]. In this study, we used the average genetic distances for determining sequence variation between the taxa studied. The availability of both standard primer sets that enable the amplification of a broad range of taxa and sequences in the database are also crucial, allowing the comparison of many species [1, 41–44]. Moreover, sequence alignment for comparison across taxa should be easy, as multiple insertions and deletions may complicate the alignment [1]. Thus, we propose four necessary properties if a genetic marker is to be used for molecular identification of parasitic helminths: (i) they must exhibit interspecific sequence variation; (ii) reference sequences in the database must be of relevant length; (3) it must be easy to align sequences across a wide phylogenetic range; and (iv) it must be easy to design universal primers.

If a marker is to be used in molecular systematic studies, two additional properties are required. First, phylogenetic analysis should be able to recover recognized higher taxa as monophyletic. In this study, we evaluated this at the order (for nematodes and cestodes) and suborder in trematodes [8, 13–15]. Secondly, the alignment used should not be saturated in terms of nucleotide substitutions [1]. Thus, for molecular systematics purposes, the six properties are: (i) the average genetic distance from order/suborder to species level; (ii) the number of monophyletic clades at the order/suborder level; (iii) adequate length of reference sequences in database; (iv) easy alignment of sequences across a wide phylogenetic range; (v) easy to design universal primers; and (vi) absence of nucleotide substitution saturation. We carried out the test for saturation using DAMBE 6 [45]. Saturation was based on the values of Iss (simple index of substitution saturation) and Iss.c (critical Iss value), with Iss < Iss.c indicating that the genetic marker was not saturated, and *vice versa *[45].

### Calculation of genetic distances and phylogenetic analyses

To calculate pairwise genetic distances for each genetic marker, we first aligned sequences for each dataset using ClustalX2.1 [46]. The aligned sequences were checked manually using Bioedit 7.0 [47]. We then calculated pairwise genetic distances using *P*-distance as the model for the aligned sequences* via* MEGA 6.0 [48]. The calculated genetic distances were categorized to derive an average distance for each taxonomical hierarchy level (order/suborder, family, genus, species). For example, at the species level, we grouped species within the same genus to obtain the average genetic distances between species and, at the genus level, grouped species in the genus that belonged to the same family to obtain the average genetic distances between the genera, and so forth. The genetic distances for each genetic marker are presented in Additional file 2: Tables S2–S11.

To obtain the number of monophyletic clades for molecular systematics and accurate phylogenetic placement for molecular identification, we conducted phylogenetic analyses using maximum likelihood (ML) and Bayesian inference (BI) algorithms. ML analysis was performed using MEGA 6.0 [48], with the best-fit nucleotide substitution model and 1000 bootstrap replicates, and BI was performed using MrBayes 3.2 [49], with four Markov chain Monte Carlo runs for 1,000,000 generations and a sampling frequency of every 100 generations. We calculated Bayesian probability values after discarding the initial 25% of phylogenetic trees as ‘burn-in.’ The phylogenetic trees generated in this study are in Additional file 3: Figures S1–S3.

### ‘K-means’ clustering and statistical analyses

We applied the unsupervised ‘K-means’ clustering machine learning algorithm implemented in Wolfram Mathematica 12.1 [50] to estimate a cut-off value for each taxonomic level using the datasets of genetic distance values. The number of clusters that we selected was pre-determined based on the taxonomic levels of the genetic distance values (e.g. four clusters represent ‘species,’ ‘genus,’ ‘family’ and ‘order’). In the ‘K-means’ method, the centroids of each cluster are initially guided by an agglomerative hierarchical algorithm, and each data point is then assigned to the nearest centroid [51, 52]. The ‘K-means’ clustering aims to partition the data points to minimize the within-cluster sum of squares in order to minimize the pairwise squared deviations of points in the same cluster until the centroids are stable [51–53]. Statistical analyses and plots were also performed using Wolfram Mathematica 12.1 [50], and the script and data used in this study for ‘K-means’ clustering analysis are available at https://github.com/slphy/Chan-HelminthMarkers.

## Results and discussion

### Assessment of suitable genetic markers for molecular systematics

Using the desirable properties described in the Materials and Methods section, we assessed the four classes of genetic markers for their suitability for application in molecular systematics of three groups of helminths and provided a guide to the genetic markers’ utility and limitations. Tables 1 and 2 summarize each class of genetic marker and its properties for molecular systematics studies; the utility and limitations of each class of genetic marker for application are listed in Additional file 4: Table S12.

**Table 1:** Properties of different classes of genetic marker in terms of their qualitative suitability for use in molecular systematics studies of helminths

Class of marker	Genetic marker	Nucleotide substitution saturation^a^	Length of references in database^b^	Easy alignment of sequences across wide phylogenetic range^b^	Easy to design universal primers^b^
Nuclear rRNA	18S rRNA	No	Mostly partial	No	Yes
	28S rRNA	No	Mostly partial	No	Yes
Nuclear spacer	ITS1	Yes	Mostly partial	No	No
	ITS2	Yes	Mostly partial	No	No
Mt protein-coding genes	*COI*	No	Complete	Yes	No
	*COII*	No	Complete	Yes	No
	*cytB*	No	Complete	Yes	No
	*NAD*1	No	Complete	Yes	No
Mt rRNA	12S rRNA	No	Complete	Yes	Yes
	16S rRNA	No	Complete	Yes	Yes

CI, Confidence interval; Iss, simple index of substitution saturation; Iss.c, critical ISS; Mt, mitochondrial; SD, standard deviation; for other abbreviations, see Abbreviation List

^a^Saturation was determined based on the sequence alignment used for each group of helminths. A ‘yes’ indicates saturation, with Iss > Iss.c

^b^Indicates the same properties used for molecular identification

**Table 2:** Properties of different classes of genetic marker in terms of their quantitative suitability for molecular systematics of helminths

Class of marker	Genetic marker	Nematodes^a^		Trematodes^a^		Cestodes^a^	
		Mean ± SD [95 CI%]	Recovered orders as monophyletic^b^	Mean ± SD [95 CI%]	Recovered suborders as monophyletic^b^	Mean ± SD [95 CI%]	Recovered orders as monophyletic^b^
Nuclear rRNA	18S rRNA	0.029* ± 0.019 [0.024–0.034]	3/6	0.036* ± 0.015 [0.033–0.038]	3/4	0.039* ± 0.021 [0.039–0.043]	4/6
	28S rRNA	0.050* ± 0.026 [0.039–0.061]	3/6	0.120* ± 0.049 [0.116–0.124]	3/4	NA	4/6
Nuclear spacer	ITS1	0.356 ± 0.227 [0.287–0.425]	0/6	0.262 ± 0.115 [0.2478–0.277]	3/4	0.546* ± 0.198 [0.481–0.612]	2/3
	ITS2	0.537* ± 0.222 [0.429–0.644]	3/6	0.171 ± 0.078 [0.160–0.181]	3/4	0.550* ± 0.106 [0.491–0.609]	5/5
Mt protein-coding	*COI*	0.215 ± 0.103 [0.197–0.234]	3/6	0.264 ± 0.047 [0.258–0.271]	2/4	0.136 ± 0.056 [0.127–0.146]	5/6
	*COII*	0.249 ± 0.139 [0.224–0.274]	4/6	0.359 ± 0.069 [0.345–0.364]	2/4	0.179 ± 0.075 [0.167–0.191]	4/5
	*cytB*	0.249 ± 0.097 [0.232–0.267]	3/6	0.259 ± 0.040 [0.254–0.265]	1/4	0.183 ± 0.080 [0.170–0.197]	5/5
	*NAD*1	0.232 ± 0.100 [0.214–0.250]	5/6	0.289 ± 0.052 [0.282–0.296]	2/4	0.193 ± 0.066 [0.982–0.203]	6/6
Mt rRNA	12S rRNA	0.198 ± 0.106 [0.178–0.217]	4/6	0.272 ± 0.055 [0.265–0.280]	2/4	0.140 ± 0.063 [0.129–0.150]	5/6
	16S rRNA	0.227 ± 0.091 [0.2109–0.244]	4/6	0.264 ± 0.051 [0.257–0.271]	3/4	0.149 ± 0.072 [0.137–0.160]	5/6

NA, No data available

*Statistically significant difference of the mean genetic distances between the markers at *P* < 0.000001), according to Kruskal–Wallis test with Dunn’s posthoc analysis

^a^Genetic distances among nematodes (Ascaridida and Spirurida), trematodes (Opisthorchiata, Echinostomata and Xiphidata), and cestodes (Taeniidae and Hymenolepididae) were used to calculate mean genetic distances

^b^The number of orders/suborders recovered as monophyletic out of the total number of recognized orders/suborders represented among available sequences (6 for nematodes, 4 for trematodes, 6 for cestodes)

#### Suitability of genetic marker based on nucleotide substitution saturation

Analysis of nucleotide substitution saturation, which is an indicator of whether a genetic marker is useful for phylogenetic inferences, in the ITS sequences chosen for investigation across the taxa sampled in this study revealed that the nuclear ribosomal ITS regions were saturated (Table 1), with Iss > Iss.c, suggesting multiple substitutions have occurred. These findings indicate that the nuclear ribosomal ITS regions are not suitable genetic markers for molecular systematics studies, particularly at higher taxonomic levels. We obtained a similar result for nematodes, with the nuclear ribosomal ITS being saturated and not useful for molecular systematics. Moreover, Thaenkham et al. [22] compared the nuclear 18S rRNA gene and the ITS2 region for Opisthorchiidae and Heterophyidae and demonstrated that compared to the 18S rRNA gene, the ITS2 region was not suitable for family-level analysis of the superfamily Opisthorchioidea. Conversely, the nuclear rRNA genes, the mitochondrial protein-coding genes and the mitochondrial rRNA genes were not saturated, with Iss < Iss.c, suggesting that they can be useful markers for inferring phylogenetic relationships.

#### Genetic distances as a measure of a genetic marker’s suitability for molecular systematics

Comparing the mean genetic distances for each marker revealed a similar trend among the three groups of helminths. As presented in Table 2, the largest genetic distances occurred in the nuclear ribosomal ITS regions of ITS1 and ITS2, suggesting that the spacer regions might not be suitable for inferring phylogenetic relationships across a broad taxonomic hierarchy. The finding is in agreement with previous studies showing that the ITS regions are not appropriate for phylogenetic comparisons between distantly related taxa [54–56]. Conversely, the mean pairwise proportion of differences in the nuclear 18S and 28S rRNA genes were the smallest, with the 18S rRNA genes having values of 0.029, 0.036 and 0.039 for nematodes, trematodes and cestodes, respectively, and the 28S rRNA genes had values of 0.050 and 0.120 for nematodes and trematodes, respectively. The mean pairwise proportion of differences among the nuclear rRNA genes was statistically different from that of all other genetic markers (*χ*^2^ = 1519.6, *df* = 9, *P* < 0.000001 for nematodes; *χ*^2^ = 581.7, *df* = 9, *P* < 0.000001 for trematodes; *χ*^2^ = 424.3, *df* = 8, *P* < 0.000001 for cestodes). The small genetic distance values of the nuclear rRNA genes can be a limiting factor and might render insufficient resolution for species-level identification.

For the mitochondrial genes, the genetic distances were significantly higher than those of the nuclear rRNA genes. Among the mitochondrial genes, the genetic distances seen in the mitochondrial rRNA genes were comparable to those in the mitochondrial protein-coding genes.

#### The number of monophyletic clades as a measure of the genetic marker’s resolution

The recovery of recognized taxa as monophyletic can also indicate the resolution of the genetic marker. The highly conserved nature of the nuclear rRNA genes makes them suitable genetic markers for molecular systematics [6]. The 18S and 28S rRNA genes have been used in the higher-level classification of nematodes, trematodes and cestodes, allowing construction of the phylogenetic framework for each group of helminths [13–15]. Our findings show that compared to other genetic markers, the nuclear rRNA genes and the mitochondrial 16S rRNA gene gave the best phylogenetic resolution for trematodes, recovering three out of four suborders as monophyletic (Table 2). For cestodes, the mitochondrial genes gave the best resolution as compared to the nuclear genes. For nematodes, the mitochondrial 12S and 16S rRNA genes exhibited the best resolution of the genetic markers (apart from *NAD*1 for nematodes), with four out of six orders as monophyletic. The mitochondrial rRNA genes are more conserved than the mitochondrial protein-coding genes, and this slightly more conserved nature has led to the mitochondrial rRNA genes being used for higher-level classification of organisms [57–59]. In helminths, the 16S rRNA gene and the nuclear rRNA genes have been used in conjunction to provide increased resolution for cestode phylogenies [60, 61]. Chan et al. also reported that the mitochondrial rRNA genes provide good resolution and can be used for molecular systematics in nematodes [59].

Thus, the results of our assessment of the genetic markers for their suitability for molecular systematics of helminths indicate that the nuclear ribosomal ITS regions might not be suitable for phylogenetic inferences at a higher taxa level due to nucleotide substitution saturation. In addition, the number of monophyletic clades obtained and sufficient genetic distances supported the resolution of the mitochondrial rRNA genes for molecular systematics, making them comparable to the commonly used nuclear rRNA genes.

### Assessment of suitable genetic markers for molecular identification

Using the four above-mentioned properties, we assessed the suitability of the genetic markers for molecular identification of nematodes, trematodes and cestodes. The results are summarized in Table 3.

**Table 3:** Properties of the different classes of genetic marker in terms of their quantitative suitability for distinguishing between species of helminths

Class of marker	Genetic marker	Nematodes^a^		Trematodes^a^		Cestodes^a^	
		Mean ± SD [95 CI%]	Closely related species^b^	Mean ± SD [95 CI%]	Closely related species^b^	Mean ± SD [95 CI%]	Closely related species^b^
Nuclear rRNA	18S rRNA	0	0.001	0.004* ± 0.002 [0.002–0.005]	0.002	0.017* ± 0.006 [0.015–0.018]	0.003
	28S rRNA	0.001 ± 0.001 [0–0.013]	0.002	0.024* ± 0.014 [0.020–0.027]	0.006	NA	NA
Nuclear spacer	ITS1	0.005 ± 0.011 [0–0.018]	0.025	0.045 ± 0.041 [0.023–0.067]	0	0.307 ± 0.283 [0.090–0.525]	0.659
	ITS2	0.117 ± 0.166 [0–1.610]	0.235	0.031 ± 0.023 [0.019–0.043]	0	0.338* ± 0.124 [0.780–1.456]	NA
Mt protein-coding	*COI*	0.026 ± 0.035 [0–0.056]	0.094	0.158 ± 0.040 [0.136–0.179]	0.089	0.085 ± 0.023 [0.079–0.090]	0.046
	*COII*	0.031 ± 0.043 [0–0.068]	0.091	0.193 ± 0.062 [0.160–0.226]	0.113	0.112 ± 0.030 [0.105–0.119]	0.029
	*cytB*	0.036 ± 0.038 [0.004–0.068]	0.166	0.174 ± 0.044 [0.151–0.198]	0.080	0.109 ± 0.028 [0.103–0.116]	0.041
	*NAD*1	0.032 ± 0.043 [0–0.068]	0.126	0.195 ± 0.058 [0.163–0.227]	0.083	0.132 ± 0.031 [0.125–0.140]	0.048
Mt rRNA	12S rRNA	0.015 ± 0.023 [0–0.035]	0.052	0.133 ± 0.045 [0.109–0.157]	0.079	0.081 ± 0.023 [0.769–0.087]	0.030
	16S rRNA	0.021 ± 0.024 [0–0.041]	0.076	0.148 ± 0.050 [0.121–0.174]	0.080	0.080 ± 0.025 [0.074–0.086]	0.024

*Statistically significant difference of mean genetic distances between the markers at *P* < 0.000001, according to Kruskal–Wallis test with Dunn’s posthoc analysis

^a^Genetic distances among nematodes (Ascaris, Parascaris, Anisakis, Toxocara and Onchocerca), trematodes (Opisthorchis, Clonorchis, Echinostoma, Fasciola, Paragonimus and Dicrocoelium) and cestodes (Taenia, Echinococcus and Hymenolepis) were used to calculate mean interspecific genetic distances

^b^Closely related species are those regarded as close sister species. If there are adequate interspecies differences between these, a marker is likely to be suitable for use in molecular identification at the species level. Examples used are *Toxocara cati vs* *T. canis* for nematodes, *Fasciola hepatica vs* *F. gigantica* for trematodes and *Taenia saginata vs* *T. asiatica* for cestodes

#### Interspecific genetic distances and phylogenetic placement as a measure for species discrimination

Sufficient sequence variation among species is an important indicator of whether the genetic marker is sufficiently robust for species discrimination [1, 8]. Interspecific genetic distance analyses across the four genetic marker classes indicated that the nuclear rRNA genes had the smallest sequence variation, with mean values that were statistically significantly different from each other (*χ*^2^ = 161.7, *df* = 9, *P* < 0.000001 for nematodes; *χ*^2^ = 124.5, *df* = 9, *P* < 0.000001 for trematodes; *χ*^2^ = 129.0, *df* = 8, *P* < 0.000001 for cestodes). For the nuclear rRNA genes, the average genetic distances between species were < 0.03, suggesting low levels of sequence variation. Moreover, for the closely related taxa, sequence variation using the 18S rRNA gene was low (0.001, 0.002 and 0.003 for nematodes, trematodes and cestodes, respectively), possibly leading to inaccurate phylogenetic placement, which is problematic in terms of species identification. Examples of this are between nematodes, such as *Toxocara canis versus* *T. cati* and *Ascaris lumbricoides versus* *A. suum*, and between trematodes, such as *Opisthorchis viverrini versus* *Clonorchis sinensis* (Additional file 3: Figures S1g and S2g). Previous studies using the 18S rRNA gene have also shown low to no sequence variation among *Trichuris* spp. and no variation between *Trichuris muris* and *T. arvicolae* [30]. Similarly, in the tapeworms, *Diphyllobothrium dentricum* and *D. ditremum*, Wicht et al. [27] demonstrated that the 18S rRNA gene had lower species discriminatory power than did the nuclear spacer regions and the mtDNA genetic markers.

Conversely, interspecific genetic distances for the nuclear ribosomal ITS spacer regions and mitochondrial genetic markers were higher than are those for the nuclear rRNA genes (except ITS1, which had lower genetic distance for nematodes). The nuclear ribosomal ITS regions tend to be used for species identification because of their faster evolution rate, resulting in highly variable sequences between species [6]. Moreover, several studies have demonstrated the effectiveness of the nuclear ribosomal ITS for the molecular identification of parasitic helminths, usually with species-specific primers, to discriminate between closely related species [10, 24, 25, 62]. For example, using the ITS1 region, Kang et al. showed that genetic distances among the closely related liver flukes were 0.045 between *O. viverrini* and *O. felineus* and 0.056 between *O. viverrini* and *C. sinensis* [62]. However, in our study, sequence variation for cestodes was unusually high (> 0.300) using the nuclear ribosomal ITS regions, perhaps due to a lack of representative sequences, thus confounding the results.

For the mitochondrial protein-coding genes, interspecific sequence variation was 0.026–0.036 for nematodes, 0.158–0.195 for trematodes and 0.085–0.132 for cestodes. Closely related species in the three groups of helminths could also be differentiated, with genetic distance values of up to 0.166 with the *cytB* gene for nematodes, 0.195 with the *NAD*1 gene for trematodes and 0.132 with the *NAD*1 gene for cestodes. This higher degree of sequence variation seen for the mitochondrial protein-coding genes compared to the nuclear rRNA genes is a clear illustration of their ability to resolve species-level relationships, even among closely related species. Consequently, it is not surprising that the mitochondrial protein-coding genes have been used widely for molecular identification, both at the species level and the population level, and to differentiate helminths from various host species [7, 26, 28, 30, 63, 64].

For the mitochondrial rRNA genes, the interspecific genetic distance values were slightly smaller than those of the mitochondrial protein-coding genes, with means of 0.015 and 0.021 for the 12S and 16S rRNA gene for nematodes, 0.133 and 0.148 for trematodes, and 0.081 and 0.080 for cestodes, respectively. However, the genetic distances were significantly higher than those for the nuclear rRNA genes, rendering the mitochondrial rRNA genes suitable for species identification. In helminths, the 12S rRNA gene has been used successfully for molecular identification, confirming the phylogenetic placement of *Setaria digitata* among filarial nematodes [65]. Moreover, Chan et al. [66] showed the suitability of the mitochondrial rRNA genes for species discrimination of closely related species in the *Angiostrongylus cantonensis* lineage.

Thus, the results of our assessment of the suitability of genetic markers for molecular identification of nematodes, trematodes and cestodes suggest that the nuclear rRNA genes might not be suitable because of low sequence variation for species discrimination. Conversely, the mtDNA genetic markers have higher sequence variation to discriminate among species and closely related species, emphasizing their suitability as markers for molecular identification.

### Advantageous properties of genetic markers for molecular systematics and identification purposes

The ease of both universal primer design and sequence alignment, in addition to the availability of full-length reference sequences, represent additional advantages that could affect a genetic marker’s suitability and utility for both molecular systematics and identification (Table 1).

First, highly conserved sequences when using the nuclear rRNA genes, as compared to the other genetic markers, can facilitate primer design that is suitable for amplifying a broad range of taxa. Universal primers for the three helminth groups have been developed using the 18S rRNA gene, and these have been used widely in molecular systematics due to their highly conserved nature [16–19]. Universal *COI* primers have also been developed and utilized for molecular-based studies [67, 68]. However, the relatively higher sequence variation in the *COI* gene in helminths compred to other groups of organisms has led to low PCR amplification success and limited taxa for analyses [42–44]. In this respect, the mitochondrial rRNA genes, being slightly less variable, possess an advantage over the more variable mitochondrial protein-coding genes and nuclear spacer regions, enabling the design of universal primer sets. Also, as compared to the more variable sequences of the mitochondrial protein-coding genes and the nuclear ribosomal ITS regions, the less variable sequences of the mitochondrial rRNA genes could increase the success of PCR amplification. Universal primers for the mitochondrial rRNA genes have been designed and utilized successfully for molecular identification and molecular systematics in nematodes [59, 66]. Secondly, the lower proportion of insertions and deletions in the sequences of the mitochondrial genetic markers enable easier sequence alignment than possible with the nuclear genetic markers. The lower proportion of indels can allow a comparison over a broader range of taxa across taxonomical levels. Lastly, with the increase in the availability of complete mitochondrial genomes in the NCBI database, full-length sequences of the mitochondrial genetic markers are readily available, presenting an advantage over the nuclear genetic markers.

Based on our evaluation of both molecular systematics and molecular identification in the selected helminths, the mitochondrial 12S and 16S rRNA genes show potential and could be suitable for applications in both contexts.

### Generation of suitable genetic distance values for future applications

To create a yardstick for guiding users when adopting genetic distances for helminths, we provide essential points to be considered and an alternative method of using genetic distances through the ‘K-means’ clustering algorithm.

#### Large genetic variation in nematodes at the same taxonomic level

A wide range of genetic distances for nematodes was observed, in contrast to trematodes and cestodes. To further investigate this observation, we selected the nuclear 18S rRNA gene, the mitochondrial 12S rRNA gene and the *COI* gene as representative genetic markers to illustrate the broad levels of genetic distances in nematodes at the same taxonomic level.

**Fig. 1:**
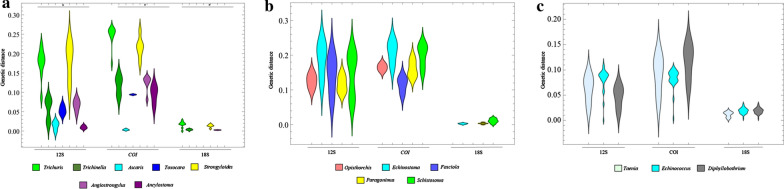
Violin-plot of genetic distances of nematodes (**a**), trematodes (**b**) and cestodes (**c**) between genera. Asterisk indicates statistically significant difference between each group, according to the Kruskal–Wallis test with Dunn’s posthoc analysis

As shown in Fig. 1a, the genetic distances between nematode genera show substantial variation, with statistically significant differences (*χ*^2^ = 39.8, *df* = 6, *P* < 0.000001). The same pattern was observed across the three genetic markers, with *Ascaris* having the smallest genetic distance and *Strongyloides* the largest. In contrast, no significant between-genus differences were found for the trematodes and cestodes (Fig. 1b, c). The same finding was also observed at the family level, where there were significant differences between nematode families (Additional file 5: Figure S4). Comparison of values at the same taxonomic level indicates a high degree of sequence variation within nematodes. Thus, our findings reveal that a general assumption of genetic distances might not be suitable and that each group of organisms should have their own genetic distance cut-off values.

#### Estimation of cut-off values per taxonomic level using the ‘K-means’ clustering algorithm

Previous studies have used genetic distances to determine whether specimens are conspecific, and in most cases, a general genetic distance value has been used as a basis for comparison [8]. In such studies, researchers mainly rely on the genetic distances of organisms that have been studied and try to find similar species to estimate whether it is a similar or different species. To circumvent this, we attempted to utilize a clustering algorithm-based machine learning strategy to estimate suitable cut-off values per taxonomic level for each genetic marker using the ‘K-means’ method and thus provide considerable data for future applications and an alternative method of analyzing genetic distances (Additional file 6: Table S13; Additional file 7: Figures S5–S7).

**Fig. 2:**
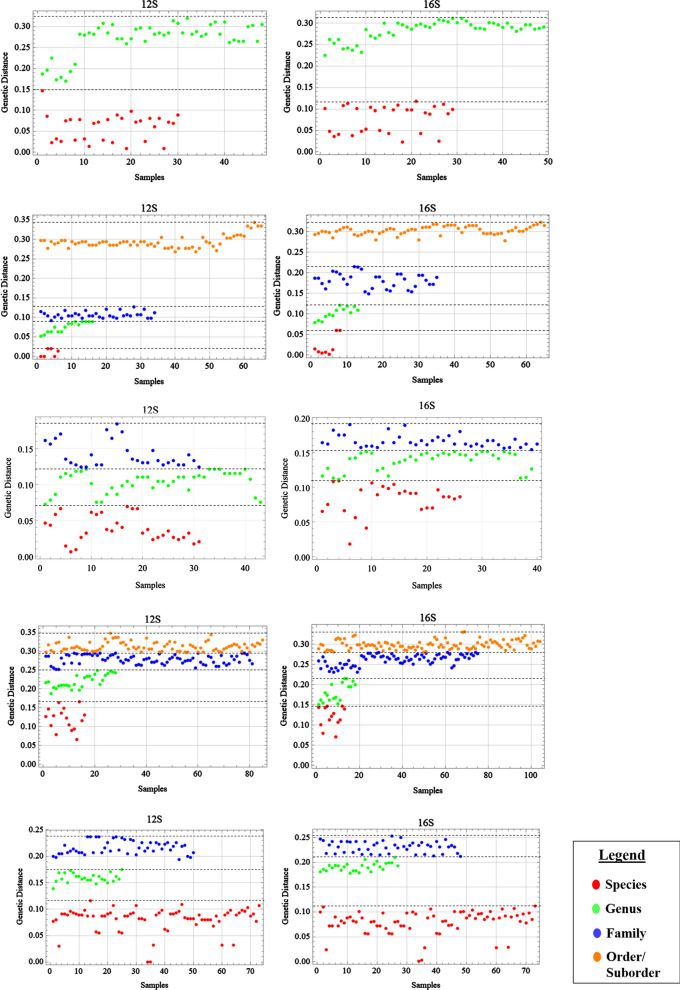
Estimated cut-off per taxonomic level of the mitochondrial rRNA genetic markers based on ‘K-means’ algorithm for nematodes belonging to Trichocephlida (**a**), nematodes belonging to Ascaridida and Spirurida (**b**), nematodes belonging to Strongylida (**c**), trematodes (**d**) and cestodes (**e**). Each colored circle indicates a genetic distance value that was input into the ‘K-means’ algorithm, and the dashed lines indicate the maximum genetic distance for each taxonomic level estimated with ‘K-means’

In our study, each taxonomic level was clearly distinguishable in the three groups of helminths for the 12S and 16S rRNA genes using the ‘K-means’ clustering algorithm, as presented in Fig. 2. Due to the large differences between each nematode order, analyses were performed separately for Trichocephalida, Ascaridida with Spirurida, and Strongylida. Similarly, the other genetic markers also showed distinct clustering patterns for each taxonomic level (Additional file 7: Figures S5–S7). The estimated cut-off values were derived from the minimum and maximum genetic distances of each cluster through the distinct clustering between each taxonomic level, allowing us to provide an estimation of the genetic distance values for each genetic marker, as provided in Additional file 6: Table S13. For example, using the 16S rRNA gene for trematodes, the estimated cut-off values between species ranged from 0.071 to 0.147, with a mean of 0.119, suggesting that the genetic distances between trematode species should fall within the specified range as estimated using the ‘K-means’ method. Likewise, for members of the same genus, the estimated cut-off values using the 16S rRNA gene for trematodes ranged from 0.151 to 0.215, with a mean of 0.181. Thus, using the ‘K-means’ clustering algorithm, we have provided a novel method for analyzing genetic distance values and generated a practical guide for future users with the estimated cut-off values per genetic marker for the helminths studied as a basis for comparison.

### Limitations

This study was limited by the availability and accuracy of the sequences in the NCBI database, which restricted the number of taxa that we could compare and analyze together across the genetic markers. Inadequate sampling can affect clade arrangement as well as the number of taxa recovered as monophyletic. Also, the species complex status for some helminth species was not considered, which could further complicate species delimitation. The results of the assessment of the genetic markers and genetic distance cut-off values were restricted to the helminth taxa that we selected, and future considerations to increase the number of species sampled should be undertaken.

## Conclusion

We have assessed the suitability of four classes of genetic marker for application in molecular systematics and molecular identification of nematodes, trematodes and cestodes. By comparing various properties and genetic distances across the taxonomic hierarchy levels, we ascertained the genetic distances for each genetic marker and showed that mitochondrial rRNA genes have the potential for utilization in molecular systematics and molecular identification of helminths. We have also revealed that following a general gauge of genetic distances might not be adequate, using evidence from the wide range of genetic distances among nematodes. In addition, we have provided a novel way of analyzing genetic distances to generate suitable cut-off values per genetic marker for each taxonomic level using the ‘K-means’ clustering algorithm. A guide to the utility and limitations of each class of genetic marker for the respective applications together with the estimated cut-off values can benefit researchers conducting molecular studies on helminths. Future research perspectives can include the use of the mitochondrial rRNA genes in molecular studies and the exploration of machine learning algorithms to aid in the classification of organisms.

## Supplementary Information

**Additional file 1: Table S1.** List of NCBI sequences used for analysis.

**Additional file 2: Tables S2–S11.** Raw genetic distances from *p*-distance analysis from MEGA for each genetic marker.

**Additional file 3: Figures S1-S3.** Phylogenetic tree for each genetic marker using maximum likelihood and Bayesian Inference algorithms.

**Additional file 4: Table S12.** General guide of the utility and limitation of each class of genetic marker for helminthes.

**Additional file 5: Figure S4.** Violin-plot of genetic distances for **a** nematodes **b** trematodes, **c** cestodes between family.

**Additional file 6: Table S13.** Estimated cut-off of each genetic marker per taxonomic level for helminths using the ‘K-means’ algorithm.

**Additional file 7: Figures S5-S7.** Plot of the estimated cut-off of each genetic marker per taxonomic level using the ‘K-means’ algorithm.

## Data Availability

All data generated during this study are included in the published article and its supplementary files.

## Abbreviations

BI: Bayesian inference
*COI*: Cytochrome *c* oxidase subunit I
*COII*: Cytochrome *c* oxidase subunit II
*cytB*: Cytochrome *b*
ITS: Internal transcribed spacer
ML: Maximum likelihood
*NAD*1: NADH dehydrogenase subunit 1
NCBI: National Center for Biotechnology Information
rRNA: Ribosomal RNA

## References

[1] Patwardhan A, Ray S, Roy A (2014). Molecular markers in phylogenetic studies—a review. J Phylogen Evolution Biol.

[2] Grover A, Sharma PC (2016). Development and use of molecular markers: past and present. Crit Rev Biotechnol.

[3] Sites J, Marshall JC (2003). Delimiting species: a renaissance issue in systematic biology. Trends Ecol Evol.

[4] Wiens JJ (2007). Species delimitation: new approaches for discovering diversity. Syst Biol.

[5] Blasco-Costa I, Cutmore SC, Milner TL, Nolan MJ (2016). Molecular approaches to trematode systematics: ‘best practice’ and implications for future study. Syst Parasitol.

[6] Hwang UW, Kim W (1999). General properties and phylogenetic utilities of nuclear ribosomal DNA and mitochondrial DNA commonly used in molecular systematics. Korean J Parasitol.

[7] Le TH, Blair D, McManus DP (2000). Mitochondrial genomes of human helminths and their use as markers in population genetics and phylogeny. Acta Trop.

[8] Blouin MS (2002). Molecular prospecting for cryptic species of nematodes: mitochondrial DNA versus internal transcribed spacer. Int J Parasitol.

[9] Allio R, Donega S, Galtier N, Nabholz B (2017). Large variation in the ratio of mitochondrial to nuclear mutation rate across animals: implications for genetic diversity and the use of mitochondrial DNA as a molecular marker. Mol Biol Evol.

[10] Choudhary K, Verma AK, Swaroop S, Agrawal N (2015). A review on the molecular characterization of digenean parasites using molecular markers with special reference to ITS region. Helminthologia.

[11] Vilas R, Criscione CD, Blouin MS (2005). A comparison between mitochondrial DNA and the ribosomal internal transcribed regions in prospecting for cryptic species of platyhelminth parasites. Parasitology.

[12] Zarlenga DS, Hoberg EP, Detwiler JT, Bruschi F (2014). Diversity and history as drivers of helminth systematics and biology. Helminth infections and their impact on global public health.

[13] Blaxter ML, De Ley P, Garey JR, Liu LX, Scheldeman P, Vierstraete A (1998). A molecular evolutionary framework for the phylum Nematoda. Nature.

[14] Olson PD, Cribb TH, Tkach VV, Bray RA, Littlewood DTJ (2003). Phylogeny and classification of Digenea (Platyhelminthes: Trematoda). Int J Parasitol.

[15] Waeschenbach A, Webster BL, Bray RA, Littlewood DTJ (2007). Added resolution among ordinal level relationships of tapeworms (Platyhelminthes: Cestoda) with complete small and large subunit nuclear ribosomal RNA genes. Mol Phylogenet Evol.

[16] Holterman M, van der Wurff A, van den Elsen S, van Megan H, Bongers T, Holovachov O (2006). Phylum-wide analysis of SSU rDNA reveals deep phylogenetic relationships among nematodes and accelerated evolution toward crown clades. Mol Biol Evol.

[17] Meldal BH, Debenham NJ, De Ley P, De Ley IT, Vanfleteren JR, Vierstraete AR (2007). An improved molecular phylogeny of the Nematoda with special emphasis on marine taxa. Mol Phylogenet Evol.

[18] Waeschenbach A, Littlewood DTJ. A molecular framework for the Cestoda. In: Caira J, Jensen K, editors. Planetary biodiversity inventory (2008–2017), tapeworms from the vertebrate bowels of the earth. Lawrence: University of Kansas, Natural History Museum; 2017. p. 431–51.

[19] Smythe AB, Holovachov O, Kocot KM (2019). Improved phylogenomic sampling of free-living nematodes enhances resolution of higher-level nematode phylogeny. BMC Evol Biol.

[20] Locker AE, Olson PD, Littlewood DTJ (2003). Utility of complete large and small subunit rRNA genes in resolving the phylogeny of the Neodermata (Platyhelminthes): implications and a review of the cercomer theory. Biol J Linn Soc.

[21] Thaenkham U, Nawa Y, Blair D, Pakdee W (2011). Confirmation of the paraphyletic relationship between families Opisthorchiidae and Heterophyidae using small and large subunit ribosomal DNA sequences. Parasitol Int.

[22] Thaenkham U, Blair D, Nawa Y, Waikagul J (2012). Families Opisthorchiidae and Heterophyidae: are they distinct?. Parasitol Int.

[23] de León GPP, Hernández-Mena D (2019). Testing the higher-level phylogenetic classification of Digenea (Platyhelminthes, Trematoda) based on nuclear rDNA sequences before entering the age of the ‘next-generation’ tree of life. J Helminthol.

[24] Maurelli MP, Rinaldi L, Capuano F, Perugini AG, Veneziano V, Cringoli G (2007). Characterization of the 28S and second internal transcribed spacer or ribosomal DNA of *Dicrocoelium dendriticum* and *Dicrocoelium hospes*. Parasitol Res.

[25] Zhao GH, Li J, Mo XH, Li XY, Lin RQ, Zou FC (2012). The second transcribed spacer rDNA sequence: an effective genetic marker for inter-species phylogenetic analysis of trematodes in the order Strigeata. Parasitol Res.

[26] Sorensen E, Drew AC, Brindley PJ, Bogh HO, Gasser RB, Qian BZ (1998). Variation in the sequence of a mitochondrial NADH dehydrogenase I gene fragment among six natural populations of *Schistosoma japonicum* from China. Int J Parasitol.

[27] Wicht B, Ruggeri-Bernardi N, Yanagida T, Nakao M, Peduzzi R, Ito A (2010). Inter- and intra-specific characterization of tapeworms of the genus *Diphyllobothrium* (Cestoda: Diphylobothriidea) from Switzerland, using nuclear and mitochondrial DNA targets. Parasitol Int.

[28] Dusitsittipon S, Criscione CD, Morand S, Komalamisra C, Thaenkham U (2017). Cryptic lineage diversity in the zoonotic pathogen *Angiostrongylus cantonensis*. Mol Phylogenet Evol.

[29] Gasser RB, Zhu X, McManus DP (1999). *NADH* dehydrogenase subunit 1 and cytochrome *c* oxidase subunit I sequences compared for members of the genus *Taenia* (Cestoda). Int J Parasitol.

[30] Callejon R, Nadler S, De Rojas M, Zurita A, Petrasova J, Cutillas C (2013). Molecular characterization and phylogeny of whipworm nematodes inferred from DNA sequences of *cox1* mtDNA and 18S rDNA. Parasitol Res.

[31] Poon RWS, Tam EWT, Lau SKP, Cheng VCC, Kwok YY, Schuster RK (2017). Molecular identification of cestodes and nematodes by *cox1* gene real-time PCR and sequencing. Diagn Microbiol Infect Dis.

[32] Sereno-Uribe AL, Gómez LA, de Núñez MO, de León GPP, García-Varela M (2019). Assessing the taxonomic validity of *Austrodiplostomum* SPP. (Digenea: Diplostomidae) through nuclear and mitochondrial data. J Parasitol.

[33] Sereno-Uribe AL, Gómez LA, de León GPP, García-Verala M (2019). Exploring the genetic diversity of *Tylodelphys* (Diesing, 1850) metacercariae in the cranial and body cavities of Mexican freshwater fishes using nuclear and mitochondrial DNA sequences, with the description of a new species. Parasitol Res.

[34] Pons J, Barraclough TG, Gomez-Zurita J, Cardoso A, Duran DP, Hazell S (2006). Sequence-based species delimitation for the DNA taxonomy of undescribed insects. Syst Biol.

[35] Zhang J, Kapli P, Pavlidis P, Stamatakis A (2013). A general species delimitation method with applications to phylogenetic placements. Bioinformatics.

[36] Yang Z, Rannala B (2014). Unguided species delimitation using DNA sequence data from multiple loci. Mol Biol Evol.

[37] Frandsen PB, Calcott B, Mayer C, Lanfear R (2015). Automatic selection of partitioning schemes for phylogenetic analyses using iterative k-means clustering of site rates. BMC Evol Biol.

[38] Yoshida R, Fukumizu K (2019). Multilocus phylogenetic analysis with gene tree clustering. Comput Biomed.

[39] Zhang S, Guo J, Wang Z. Combing [sic] K-means clustering and local weighted maximum discriminant projections for weed species recognition. Front Comput Sci. 2019;1.

[40] McManus DP, Bowles J (1996). Molecular genetic approaches to parasite identification: their value in diagnostic parasitology and systematics. Int J Parasitol.

[41] Ghatani S, Shylla JA, Roy B, Tandon V (2014). Multilocus sequence evaluation for differentiating species of the trematode family Gastrothylacidae, with a note on the utility of mitochondrial *COI* motifs in species identification. Gene.

[42] Moszczynska A, Locke SA, McLaughlin JD, Marcogliese DJ, Crease TJ (2009). Development of primers for the mitochondrial cytochrome *c* oxidase I gene in digenetic trematodes (Platyhelminthes) illustrates the challenge of barcoding parasitic helminths. Mol Ecol Res.

[43] Creer S, Fonseca VG, Porazinska DL, Giblin-Davis RM, Sung W, Power DM (2010). Ultrasequencing of the meiofaunal biosphere: practice, pitfalls and promises. Mol Ecol Res.

[44] Andujar C, Arribas P, Yu DW, Vogler AP, Emerson BC (2018). Why the *COI* barcode should be the community DNA metabarcode for the metazoa. Mol Ecol Res.

[45] Xia X (2017). DAMBE 6: new tools for microbial genomics, phylogenetics and molecular evolution. J Hered.

[46] Thompson J, Gibson T, Higgins D. Multiple sequence alignment using ClustalW and ClustalX. Curr Protoc Bioinformatics. 2002; Chapter 2: Unit 2.3 10.1002/0471250953.bi0203s00 18792934

[47] Hall T (1999). BioEdit: a user-friendly biological sequence alignment editor and analysis program for Windows 95/98/NT. Nucleic Acids Symp Ser.

[48] Tamura K, Stecher G, Peterson D, Filipski A, Kumar S (2013). MEGA 6: molecular evolutionary genetic analysis version 6.0. Mol Biol Evol.

[49] Huelsenbeck JP, Ronquist F (2001). MRBAYES: Bayesian inference of phylogenetic trees. Bioinformatics.

[50] Wolfram Research Inc. Mathematica version 12.1. Champaign: Wolfram Research, Inc.; 2020.

[51] Faber V. Clustering and the continuous k-means algorithm. Los Alamos Sci. 1994;22:138–44.

[52] Morisette L, Chartier S (2013). The k-means clustering technique: general considerations and implementation in Mathematica. Tutor Quant Methods Psychol.

[53] Sangkaew S, Tan LK, Ng LC, Ferguson NM, Dorigatti I (2020). Using cluster analysis to reconstruct dengue exposure from cross-sectional serological studies in Singapore. Parasites Vectors.

[54] Nolan MJ, Cribb TH (2005). The use and implications of ribosomal DNA sequencing for the discrimination of digenean species. Adv Parasitol.

[55] Le TH, Nguyen KT, Nguyen NTB, Doan HTT, Dung DT, Blair D (2017). The ribosomal transcription units of *Haplorchis pumilio* and *H. taichui* and the use of 28S rDNA sequences for phylogenetic identification of common heterophyids in Vietnam. Parasites Vectors.

[56] Le TH, Pham KTL, Doan HTT, Le Xuyen TK, Nguyen KT, Lawton SP (2020). Description and phylogenetic analyses of ribosomal transcription units from species of Fasciolidae (Platyhelminthes: Digenea). J Helminthol.

[57] Vences M, Thomas M, van der Meijden A, Chiari Y, Vietes DR (2005). Comparative performance of the 16S rRNA gene in DNA barcoding of amphibians. Front Zool.

[58] Yang L, Tan Z, Wang D, Xue L, Guan M, Huang T (2014). Species identification through mitochondrial rRNA genetic analysis. Sci Rep.

[59] Chan AHE, Chaisiri K, Morand S, Saralamba N, Thaenkham U (2020). Evaluation and utility of mitochondrial ribosomal genes for molecular systematics of parasitic nematodes. Parasites Vectors.

[60] Littlewood DTJ, Waeschenbach A, Nikolov PN (2008). In search of mitochondrial markers for resolving the phylogeny of cyclophyllidean tapeworms (Platyhelminthes, Cestoda)—a test study with Davaineidae. Acta Parasitol.

[61] Waeschenbach A, Webster B, Littlewood DTJ (2012). Adding resolution to ordinal level relationships of tapeworms (Platyhelminthes: Cestoda) with large fragments of mtDNA. Mol Phylogenet Evol.

[62] Kang S, Sultana T, Loktev VB, Wongratanacheewin S, Sohn WM, Eom KS (2008). Molecular identification and phylogenetic analysis of nuclear rDNA sequences among three opisthorchid liver flukes (Opisthorchiidae: Trematoda). Parasitol Int.

[63] Zarowiecki MZ, Huyse T, Littlewood DTJ (2007). Making the most of mitochondrial genomes—markers for phylogeny, molecular ecology and barcodes in *Schistosoma* (Platyhelminthes: Digenea). Int J Parasitol.

[64] Rezabkova L, Brabec J, Jirku M, Dellerba M, Kuchta R, Modry D (2019). Genetic diversity of the potentially therapeutic tapeworm *Hymenolepis diminuta* (Cestoda: Cyclophyllidea). Parasitol Int.

[65] Yatawara L, Wickramasinghe S, Nagataki M, Rajapakse RPVJ, Agatsuma T (2007). Molecular characterization and phylogenetic analysis of *Setaria digitata* of Sri Lanka based on *COI* and 12S rDNA genes. Vet Parasitol.

[66] Chan AHE, Chaisiri K, Dusitsittipon S, Jakkul W, Charoennitiwat V, Komalamisra C (2020). Mitochondrial ribosomal genes as novel genetic markers for discrimination of closely related species in the *Angiostrongylus cantonensis* lineage. Acta Trop.

[67] Folmer O, Black M, Hoeh W, Lutz R, Vrijenhoek R (1994). DNA primers for amplification of mitochondrial cytochrome *c* oxidase subunit I from diverse metazoan invertebrates. Mol Mar Biol Biotechnol.

[68] Bowles J, Blair D, McManus DP (1992). Genetic variants within the genus *Echinococcus* identified by mitochondrial DNA sequencing. Mol Biochem Parasitol.

