# Supplementary material for: Assessing the suitability of mitochondrial and nuclear DNA genetic markers for molecular systematics and species identification of helminths
Source: Parasit Vectors. 2021 May 1;14:233. doi: 10.1186/s13071-021-04737-y (PMC8088577; doi:10.1186/s13071-021-04737-y)
Supplement: Supplementary file 1 — Additional file 1: Table S1. List of NCBI sequences used for analysis. [file 13071_2021_4737_MOESM1_ESM.docx]

**Additional file 1: Table S1.** List of NCBI sequences used for analysis

| **Group** | **Order/Suborder** | **Family** | **Species** | **NCBI accession number** | | | | |
| --- | --- | --- | --- | --- | --- | --- | --- | --- |
|  |  |  |  | **Mt genome** | **18S** | **28S** | **ITS1** | **ITS2** |
| Cestoda | Pseudophyllidea | Diphyllobothriidae | *Diphyllobothrium latum* | DQ985706 | KF218251 | KY552817 | - | KF218251 |
|  |  |  | *Diphyllobothrium latum* | AB269325 | DQ768156 | DQ925326 | - | - |
|  |  |  | *Diphyllobothrium nihonkaiense* | EF420138 | AB512013 | LC312467 | AB288368 | AB288368 |
|  |  |  | *Diphyllobothrium nihonkaiense* | AB268585 | AM408316 | - | - | - |
|  |  |  | *Diphyllobothrium stemmacephalum* | AP017648 | AF124459 | KY552825 | - | - |
|  |  |  | *Diphyllobothrium stemmacephalum* | - | KY552793 | - | - | - |
|  |  |  | *Diphyllobothrium dentriticum* | - | - | - | JN153018 | - |
|  |  |  | *Diphyllobothrium sp* | - | - | - | - | KY945917 |
|  |  |  | *Spirometra erinaceieuropaei* | KY114889 | KY552802 | KY552835 | - | - |
|  |  |  | *Spirometra erinaceieuropaei* | KJ599680 | D64072 | - | - | - |
|  |  |  | *Spirometra erinaceieuropaei* | KU852381 | - | - | - | - |
|  |  |  | *Spirometra decipiens* | KJ599679 | - | - | - | - |
|  |  |  | *Schistocephalus solidus* | AP017669 | AF124460 | - | - | - |
|  | Cyclophylidea | Taeniidae | *Taenia solium* | AB086256 | AB731615 | **-** | KJ207061 | - |
|  |  |  | *Taenia solium* | - | GQ260091 | - | - | - |
|  |  |  | *Taenia saginata* | AY684274 | JQ609338 | **-** | AY392045 | AY954520 |
|  |  |  | *Taenia saginata* | - | AB731616 | - | - | - |
|  |  |  | *Taenia asiatica* | AF445798 | AB731617 | **-** | KJ207075 | - |
|  |  |  | *Taenia asiatica* | - | GQ260088 | **-** | AY392046 | - |
|  |  |  | *Taenia crocutae* | AB905201 | - | - | - | - |
|  |  |  | *Taenia multiceps* | GQ228818 | AB731621 | - | - | - |
|  |  |  | *Taenia multiceps* | - | GQ260089 | - | - | - |
|  |  |  | *Taenia arctos* | AB905199 | - | - | - | - |
|  |  |  | *Taenia regis* | AB905198 | - | - | - | - |
|  |  |  | *Taenia pisiformis* | GU569096 | JQ609339 | **-** | JX317672 | JX317669 |
|  |  |  | *Taenia hydatigena* | GQ228819 | AB731619 | - | - | - |
|  |  |  | *Taenia hydatigena* | FJ518620 | GQ260090 | - | - | - |
|  |  |  | *Taenia crassiceps* | AF216699 | AB731618 | - | - | - |
|  |  |  | *Taenia taeniaefo* | FJ597547 | JQ609340 | - | - | - |
|  |  |  | *Taenia taeniaefo* |  | AB731629 | - | - | - |
|  |  |  | *Echinococcus multilocularis* | AB018440 | AB731634 | **-** | AJ237778 | - |
|  |  |  | *Echinococcus granulosus* | AF297617 | AB731639 | **-** | AF132701 | KR872308 |
|  |  |  | *Echinococcus granulosus* | MK774655 | GQ260092 | - | - | - |
|  |  |  | *Echinococcus granulosus* | MH301022 | - | - | - | - |
|  |  |  | *Echinococcus granulosus* | KJ559023 | - | - | - | - |
|  |  |  | *Echinococcus equinus* | AF346403 | AB731640 | - | - | - |
|  |  |  | *Echinococcus equinus* | AB786665 | - | - | - | - |
|  |  |  | *Echinococcus canadensis* | AB208063 | AB731642 | **-** | - | - |
|  |  |  | *Echinococcus canadensis* | MN340039 | - | - | KP866147 | - |
|  |  |  | *Echinococcus ortleppi* | AB235846 | AB731641 | - | - | - |
|  |  |  | *Echinococcus vogeli* | AB208546 | AB731636 | - | - | - |
|  |  |  | *Echinococcus shiquicus* | AB208064 | AB731635 | - | - | - |
|  |  |  | *Echinococcus oligarthrus* | AB208545 | AB731637 | - | - | - |
|  |  | Hymenolepididae | *Hymenolepis diminuta* | AF314223 | JX310720 | HM138522 | AF461125 | AF461125 |
|  |  |  | *Hymenolepis diminuta* | - | AF124475 | - | - | - |
|  |  |  | *Hymenolepis diminuta* | - | - | KT026970 | - | - |
|  |  |  | *Hymenolepis nana* | KT951722 | AY193875 | KU748351 | AF461124 | AF461124 |
|  |  |  | *Hymenolepis nana* | - | - | LC064145 | - | - |
|  |  | Dilepididae | *Dipylidium caninum* | MG587892 | AB731643 | - | AM491339 | AM491339 |
|  | Caryophyllidea | Capingentidae | *Breviscolex orientalis* | KY486752 | AF286978 | JQ034117 | - | - |
|  |  | Caryophyllaeidae | *Caryophyllaeus brachycollis* | KT028770 | JQ034137 | JQ034120 | - | KF700249 |
|  | Nippotaeniidea | Nippotaeniidae | *Nippotaenia chaenogobii* | JQ268550 | AF286987 | AF286933 | - | - |
|  | Proteocephalidea | Proteocephalidae | *Gangesia oligochis* | MF314173 | - | JX477451 | - | - |
|  |  |  | *Gangesia parasiluri* | - | AF267293 | - | - | AY551144 |
|  | Tetraphyllidea | Phyllobothriidae | *Clistobothrium montaukensis* | JQ268541 | AF286996 | LC195134 | - | - |
|  |  |  | *Clistobothrium sp.* | - | - | - | KU724058 | KU724058 |
|  |  | Rhinebothriidae | *Rhinebothrium reydai* | MK896864 | - | - | - | - |
|  |  |  | *Rhinebothrium maccallumi* | - | AF124476 | AF286962 | - | - |
| Trematoda | Opisthorchiata | Opisthorchiidae | *Opisthorchis felinius* | EU921260 | - | - | EU038134 | EF688142 |
|  |  |  | *Opisthorchis viverrini* | JF739555 | JF823987 | JF823990 | EU038153 | MG797539 |
|  |  |  | *Clonorchis sinensis* | FJ381664 | AF229852 | JF823989 | JQ048576 | MF319653 |
|  |  |  | *Amphimerus sp.* | MK238506 | - | - | MK238505 | MK238505 |
|  |  |  | *Amphimerus ovalis* | - | AY222121 | AY116876 | - | - |
|  |  | Heterophyidae | *Haplorchis taichui* | KF214770 | HM004213 | KX815126 | KX815126 | KX815126 |
|  |  |  | *Haplorchis pumilo* | - | HM004196 | KX815125 | KX815125 | KX815125 |
|  |  |  | *Metagonimus yokogawai* | KC330755 | HM004208 | HQ832641 | KJ631740 | HM004158 |
|  |  |  | *Metagonimus takahashii* | - | - | HQ832638 | - | - |
|  |  |  | *Metagonimus miyatai* | - | - | HQ832635 | - | - |
|  |  |  | *Metagonimus suifunensis* | - | - | KX387460 | KX387524 | KX387524 |
|  |  |  | *Metagonimus pusillus* | - | - | - | MF407173 | - |
|  | Xiphidata | Paragonimidae | *Paragonimus westermani* | AF219379 | AY222140 | HM172631 | AF040935 | JN656204 |
|  |  |  | *Paragonimus heterotremus* | MH059809 | LT855188 | HM172617 | - | - |
|  |  |  | *Paragonimus pseudoheterotremus* | - | HM004210 | - | - | - |
|  |  |  | *Paragonimus ohirai* | KX765277 | - | HM172621 | AF040931 | - |
|  |  |  | *Paragonimus kellicotti* | MH322000 | HQ900670 | - | HQ900670 | HQ900670 |
|  |  |  | *Paragonimus siamensis* | - | - | HM172624 | - | JQ322636 |
|  |  |  | *Paragonimus miyazakii* | - | - | HM172620 | - | - |
|  |  |  | *Paragonimus mexicanus* | - | - | HM172619 | - | - |
|  |  |  | *Paragonimus macrochis* | - | - | HM172618 | - | - |
|  |  |  | *Paragonimus harinasutai* | - | - | HM172616 | - | - |
|  |  | Dicrocoeliidae | *Dicrocoelium dentriticum* | KF318787 | Y11236 | AF151939 | KC774524 | KC774524 |
|  |  |  | *Dicrocoelium chinensis* | KF318786 | - | - | KF734795 | KF734795 |
|  |  |  | *Dicrocoelium orientalis* | - | EF547131 | - | - | - |
|  |  |  | *Dicrocoelium hospes* | - | - | AY251233 | - | - |
|  |  |  | *Lyperosomum longicauda* | MK685274 | - | MK685270 | - | - |
|  |  |  | *Lyperosomum collurionis* | - | AY222143 | - | - | - |
|  |  |  | *Lyperosomum collurionis* | - | - | KU212193 | - | - |
|  | Diplostomata | Schistosomatidae | *Schistosoma haematobium* | DQ157222 | - | AY157263 | GU257398 | GU257398 |
|  |  |  | *Schistosoma kisumuensis* | - | - | FJ897154 | - | - |
|  |  |  | *Schistosoma japonicum* | KU196417 | AY157226 | Z46504 | FJ852573 | FJ852573 |
|  |  |  | *Schistosoma mansoni* | - | U65657 | - | KX011042 | KX011042 |
|  |  |  | *Schistosoma mekongi* | AF217449 | AY157228 | - | U89871 | - |
|  |  |  | *Schistosoma indicum* | MN637821 | - | LC224107 | - | - |
|  |  |  | *Schistosoma spindale* | DQ157223 | - | LC224106 | - | - |
|  |  |  | *Schistosoma intercalatum* | - | DQ354363 | DQ354362 | - | - |
|  |  |  | *Schistosoma incognitum* | - | AY157229 | - | - | - |
|  |  |  | *Schistosoma bovis* | - | AY318828 | - | - | - |
|  |  |  | *Schistosoma mattheei* | - | AY157237 | - | - | - |
|  |  |  | *Schistosoma nasale* | - | - | KR423864 | - | - |
|  |  | Diplostomaidae | *Diplostomum ardeae* | MT259035 | - | - | MT259036 | MT259036 |
|  |  |  | *Diplostomum phoxini* | - | AY222090 | AY222173 | - | - |
|  |  |  | *Diplostomum spathaceum* | - | AY245761 | - | - | - |
|  |  |  | *Diplostomum compactum* | - | AY245764 | - | - | - |
|  |  |  | *Diplostomum pseudospathace* | - | - | - | KR269766 | - |
|  |  |  | *Diplostomum huronense* | - | - | - | - | AY123044 |
|  |  |  | *Diplostomum indistinctum* | - | - | - | - | AY123043 |
|  |  | Clinostomidae | *Clinostomum complanatum* | KM923964 | - | - | JF718629 | - |
|  |  |  | *Clinostomum tataxumi* | - | MF398349 | MF398321 | - | - |
|  |  |  | *Clinostomum marginatum* | - | MF398350 | MF398323 | JN108032 | JF718633 |
|  | Echinostomata | Echinostomatidae | *Echinostoma revolutum* | MN116706 | AY222132 | KP065598 | - | AF067850 |
|  |  |  | *Echinostoma miyagawai* | MN116740 | - | KY436408 | - | MH796365 |
|  |  |  | *Echinostoma hortense* | KR062182 | - | - | - | - |
|  |  |  | *Echinostoma paraensei* | - | FJ380226 | - | AF336234 | AF336234 |
|  |  |  | *Echinostoma caproni* | - | L06567 | - | - | - |
|  |  | Fasciolidae | *Fasciola hepatica* | AF216697 | AJ004969 | AY222244 | MN970007 | MN970007 |
|  |  |  | *Fasciola gigantica* | KF543342 | AJ011942 | AY222245 | MN970008 | - |
|  |  |  | *Fasciola jacksoni* | KX787886 | - | EU025871 | MN970006 | MN970006 |
|  |  |  | *Fasciolopsis buski* | KX169163 | L06668 | - | EF612477 | - |
|  |  |  | *Fasciolopsis buski* | - | AY311386 | - | - | - |
| Nematoda | Trichocephalida | Trichuridae | *Trichuris trichiura* | KT449826 | AB699091 | - | GQ301555 | GQ301555 |
|  |  |  | *Trichuris suis* | KT449823 | HF586905 | - | AM993016 | AM993014 |
|  |  |  | *Trichuris ovis* | NC018597 | HF586911 | - | JF680987 | JF680987 |
|  |  |  | *Trichuris discolor* | NC018596 | HF586910 | - | KT630810 | KT630810 |
|  |  |  | *Trichuris muris* | NC028621 | HF586907 | - | - | KU575094 |
|  |  | Trichinellidae | *Trichinella patagoniensis* | KM357412 | MF628272 | - | - | - |
|  |  |  | *Trichinella spiralis* | NC002681 | U60231 | AF342803 | KC006415 | KC006415 |
|  |  |  | *Trichinella murrelli* | NC025751 | AY851259 | - | KC006408 | KC006410 |
|  |  |  | *Trichinella nelsoni* | NC025753 | AY851261 | - | - | - |
|  |  |  | *Trichinella zimbabwensis* | KM357421 | AY851264 | - | - | - |
|  |  |  | *Trichinella pseudospiralis* | NC025749 | AY851258 | - | - | - |
|  |  |  | *Trichinella britovi* | NC025750 | AY851257 | - | - | - |
|  |  |  | *Trichinella papuae* | M357417 | AY851263 | - | - | - |
|  | Oxyurida | Oxyuridae | *Enterobius vermicularis* | EU281143 | HQ646164 | - | HQ646164 | HQ646164 |
|  |  |  | *Oxyuris equi* | NC027190 | KU180664 | - | - | - |
|  |  |  | *Oxyuris equi* | - | - | - | - | - |
|  |  |  | *Oxyuris equi* | - | - | EF180062 | - | - |
|  |  |  | *Oxyuris equi* | - | - | KU180664 | - | - |
|  |  |  | *Aspiculuris tetraptera* | KT764937 | MH215350 | - | - | - |
|  |  |  | *Syphacia obvelata* | KT900946 | EF464554 | EF464554 | - | - |
|  |  |  | *Syphacia muris* | AP017697 | EF464553 | EF464553 | - | - |
|  | Ascaridida | Ascarididae | *Parascaris univalens* | KM067271 |  | - | - | - |
|  |  |  | *Parascaris equorum* | MF678786 | U94378 | - | MF678787 | MF678787 |
|  |  |  | *Ascaris suum* | KY045805 | U94367 | - | MF358950 | MF358962 |
|  |  |  | *Ascaris suum* | - | - | - | - | - |
|  |  |  | *Ascaris suum* | - | - | U94367 | - | - |
|  |  |  | *Ascaris ovis* | NC036666 | - | - | KU522455 | KU522455 |
|  |  |  | *Ascaris lumbricoides* | HQ704900 | U94366 |  | MF358963 | MF358961 |
|  |  |  | *Ascaris sp.* | - | - | M58348 | - | - |
|  |  | Toxocaridae | *Toxocara cati* | AM411622 | EF180059 | JN256975 | JF837173 | JF837173 |
|  |  |  | *Toxocara canis* | AM411108 | U94382 | JN256976 | JF837169 | JF837169 |
|  |  |  | *Toxocara malaysiensis* | AM412316 | - | - | - | - |
|  |  |  | *Toxocara vitulorum* | - | - | KJ398347 | - | - |
|  |  | Anisakidae | *Anisakis simplex* | AY994157 | MF072711 | - | GU735486 | - |
|  |  |  | *Anisakis pegreffii* | NC034329 | EF180082 | EF180082 | KF512856 | - |
|  |  |  | *Anisakis pegreffii* | - | - | MF072697 | - | - |
|  |  |  | *Anisakis sp.* | - | - | U94365 | - | - |
|  |  |  | *Contracaecum rudolphii* | FJ905109 | - | - | - | - |
|  |  | Heterakidae | *Heterakis gallinarum* | KU529973 | DQ503462 | DQ503462 | - | AJ876758 |
|  |  |  | *Heterakis gallinarum* | - | - | - | - | AJ876757 |
|  |  |  | *Heterakis gallinarum* | - | - | - | - | AJ007454 |
|  |  |  | *Heterakis beramporia* | NC029838 | - | - | - | - |
|  |  |  | *Heterakis dispar* | NC042411 | - | - | - | - |
|  |  |  | *Heterakis spumosa* | - | - | MH571872 | JX845278 | JX845278 |
|  |  |  | *Heterakis spumosa* | - | - | - | - | MH571868 |
|  |  |  | *Heterakis spumosa* | - | - | - | - | MH571868 |
|  |  |  | *Heterakis isolonche* | - | - | - | KM212953 | - |
|  |  |  | *Heterakis sp.* | - | - | AF083003 | - | - |
|  | Spirurida | Onchocercidae | *Onchocerca volvulus* | NC001861 | - | - | AF228573 | AF228574 |
|  |  |  | *Onchocerca volvulus* | - | - | - | - | DQ317666 |
|  |  |  | *Onchocerca volvulus* | - | - | - | - | EU272179 |
|  |  |  | *Onchocerca ochengi* | KX181290 | - | - | - | - |
|  |  |  | *Onchocerca flexuosa* | HQ214004 | - | - | - | - |
|  |  |  | *Loa loa* | HQ186250 | XR002251421 | - | EU272176 | - |
|  |  |  | *Wuchereria bancrofti* | JQ316200 | AY843438 | AY843438 | AY621478 | - |
|  |  |  | *Wuchereria bancrofti* | - | - | AY843437 | - | - |
|  |  |  | *Wuchereria bancrofti* | - | - | AF227234 | - | - |
|  |  |  | *Dirofilaria immitis* | AJ537512 | AF182647 | - | - | - |
|  |  | Dracunculidae | *Dracunculus medinensis* | JN555591 | AY947720 | - | - | - |
|  |  | Gnathostomatidae | *Gnathostoma doloresi* | NC032073 | - | - | AB181156 | AB181156 |
|  |  |  | *Gnathostoma nipponicum* | KX826911 | - | - | AB181157 | AB181157 |
|  |  |  | *Gnathostoma spinigerum* | NC027726 | - | - | AB181155 | KP784333 |
|  |  |  | *Gnathostoma binucleatum* | - | Z96946 | - | - | - |
|  |  |  | *Gnathostoma turgidum* | - | Z96948 | - | - | - |
|  |  |  | *Gnathostoma neoprocyonis* | - | Z96947 | - | - | - |
|  |  | Gongylonematidae | *Gongylonema pulchrum* | NC026687 | AB495389 | AB495401 | - | - |
|  | Rhabditida | Strongyloididae | *Strongyloides papillosus* | NC028622 | AB923886 | - | - | - |
|  |  |  | *Strongyloides ratti* | NC028623 | AB923889 | AB453329 | U43580 | - |
|  |  |  | *Strongyloides ratti* | - | - | AB453328 | - | - |
|  |  |  | *Strongyloides ratti* | - | - | AB923889 | - | - |
|  |  |  | *Strongyloides venezuelensis* | NC028229 | AB923887 | - | - | - |
|  |  |  | *Strongyloides stercoralis* | LC050212 | AB923888 | - | JX489153 | EF653265 |
|  |  |  | *Parastrongyloides trichosuri* | LC050209 | AB923885 | - | - | - |
|  | Strongylida | Trichostrongylidae | *Haemonchus placei* | NC029736 | - | - | - | - |
|  |  |  | *Haemonchus contortus* | EU346694 | - | EU086375 | - | - |
|  |  |  | *Haemonchus sp.* |  | - | DQ503465 | - | - |
|  |  |  | *Nippostrongylus brasiliensis* | NC033886 | AJ920356 | - | - | - |
|  |  | Metastrongylidae | *Angiostrongylus malaysiensis* | NC030332 | EF514914 | - | - | - |
|  |  |  | *Angiostrongylus costaricensis* | GQ398122 | KX378964 | EF514913 | GU587746 | - |
|  |  |  | *Angiostrongylus cantonensis* | GQ398121 | AY295804 | - | GU587760 | JQ806760 |
|  |  |  | *Angiostrongylus vasorum* | NC018602 | AJ920365 | KY654093 | GU733324 | EU915248 |
|  |  |  | *Angiostrongylus mackerrasae* | MN793157 | - | - | - | - |
|  |  | Ancylostomatidae | *Necator americanus* | AJ417719 | AJ920348 | AY295811 | KX577786 | KM891738 |
|  |  |  | *Ancylostoma duodenale* | AJ417718 | EU344798 | - | EU344797 | MG271919 |
|  |  |  | *Ancylostoma ceylanicum* | NC035142 | - | - | KM066110 | KM066110 |
|  |  |  | *Ancylostoma caninum* | - | AJ920347 | - | - | - |
|  |  |  | *Ancylostoma tubaeforme* | NC034289 | - | - | JQ812691 | JQ812691 |
|  |  | Strongylidae | *Oesophagostomum quadrispinulatum* | FM161883 | - | - | - | - |
|  |  |  | *Oesophagostomum dentatum* | FM161882 | - | - | - | - |
|  |  |  | *Strongylus vulgaris* | GQ888717 | - | - | - | - |
|  |  |  | *Strongylus equinus* | NC026868 | - | - | - | - |
