# Supplementary material for: Assessing the suitability of mitochondrial and nuclear DNA genetic markers for molecular systematics and species identification of helminths
Source: Parasit Vectors. 2021 May 1;14:233. doi: 10.1186/s13071-021-04737-y (PMC8088577; doi:10.1186/s13071-021-04737-y)
Supplement: Supplementary file 3 — Additional file 3: Figures S1-S3. Phylogenetic tree for each genetic marker using maximum likelihood and Bayesian Inference algorithms. [file 13071_2021_4737_MOESM3_ESM.docx]

**Additional file 3: Figure S1 to S3.** Phylogenetic trees of helminths for each genetic marker inferred using ML and BI algorithms


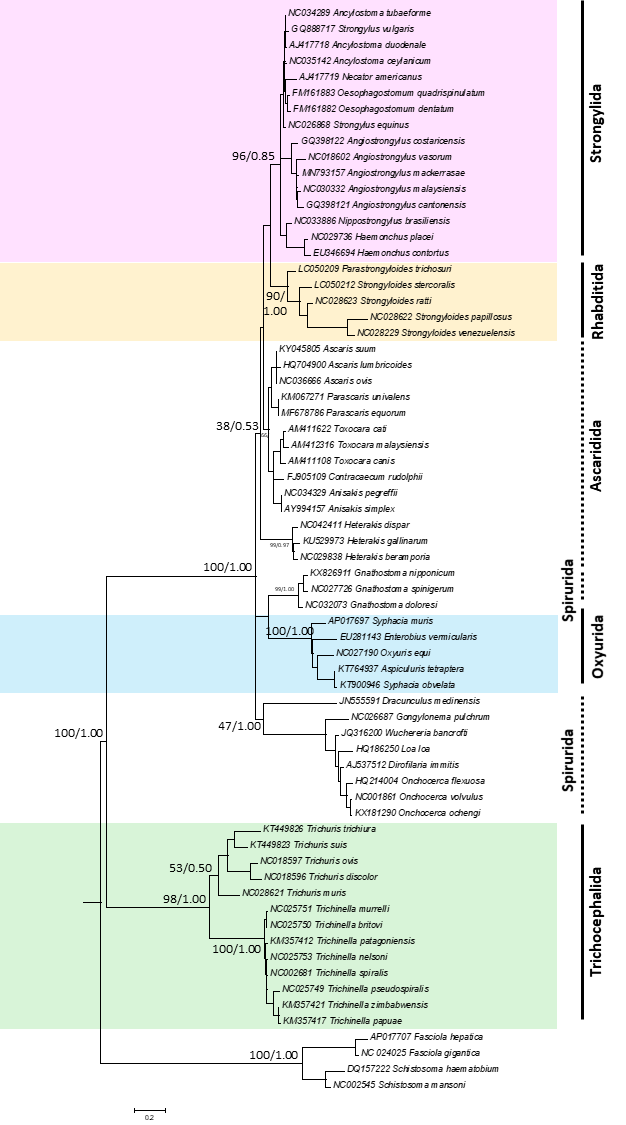


**Fig S1a. Phylogeny using 12S rRNA gene sequences as genetic marker for nematodes.**

Phylogenetic analyses was inferred using maximum likelihood (GTR+G) and Bayesian inference. Numbers at nodes indicate BS/PP values. Clades that are monophyletic at the order level are shaded and indicated with a continuous line.


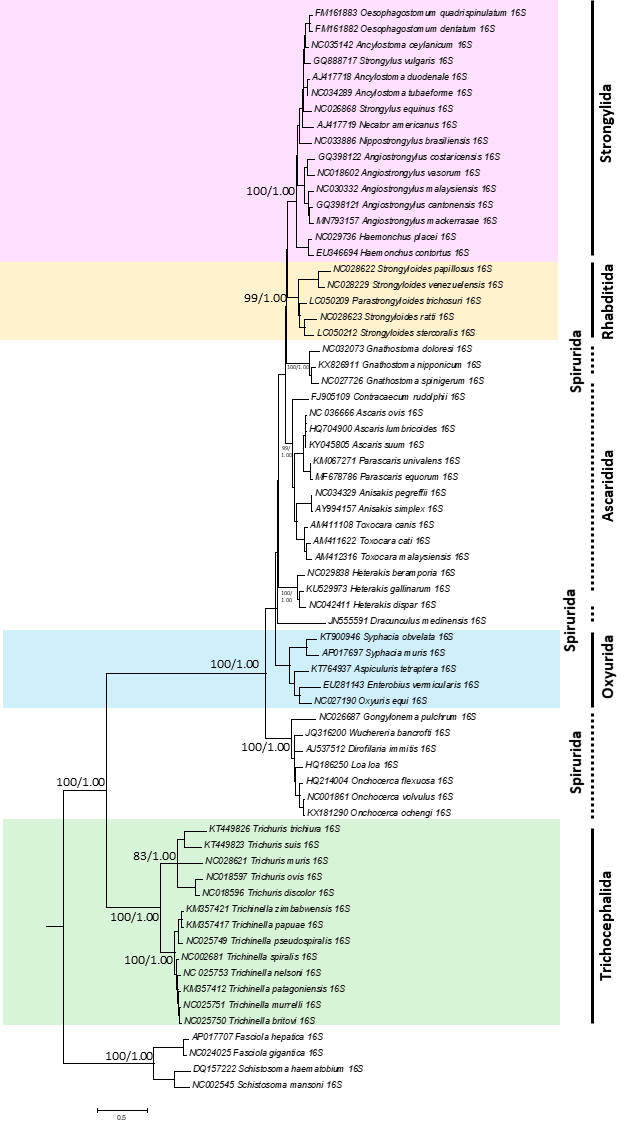


**Fig S1b. Phylogeny using 16S rRNA gene sequences as genetic marker for nematodes.**

Phylogenetic analyses was inferred using maximum likelihood (GTR+G+I) and Bayesian inference. Numbers at nodes indicate BS/PP values. Clades that are monophyletic at the order level are shaded and indicated with a continuous line.


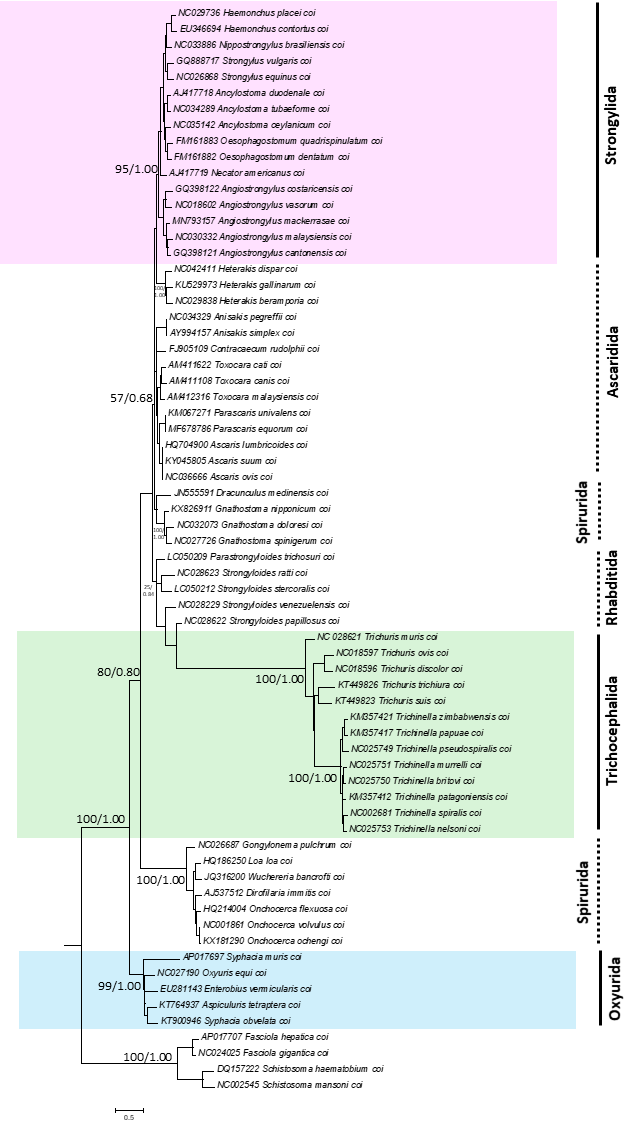


**Fig S1c. Phylogeny using *COI* gene sequences as genetic marker for nematodes.**

Phylogenetic analyses was inferred using maximum likelihood (GTR+G+I) and Bayesian inference. Numbers at nodes indicate BS/PP values. Clades that are monophyletic at the order level are shaded and indicated with a continuous line.


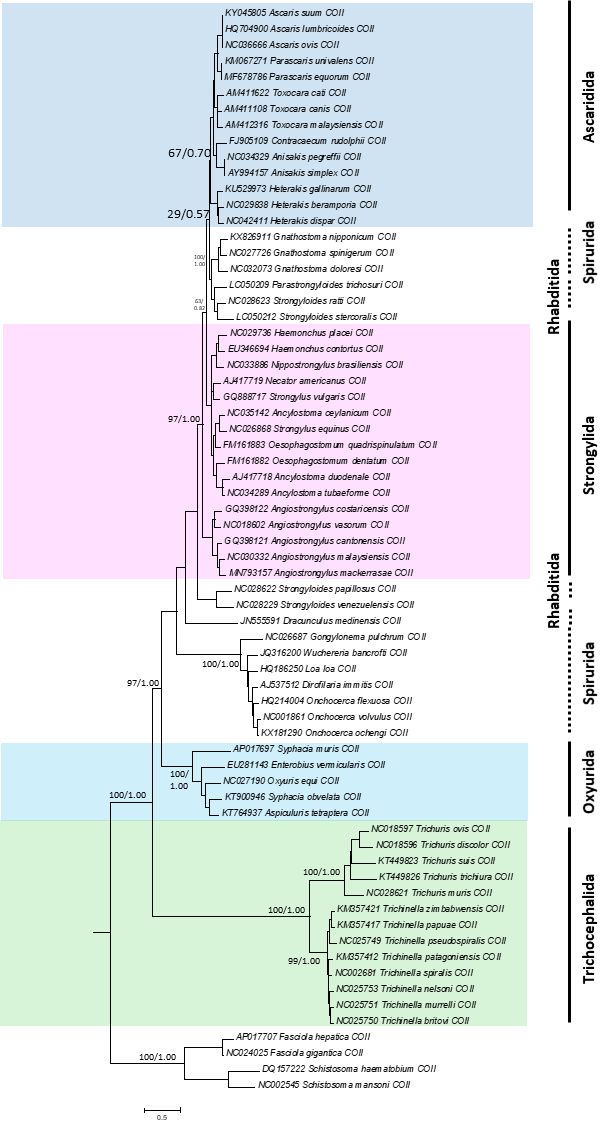


**Fig S1d. Phylogeny using *COII* gene sequences as genetic marker for nematodes.**

Phylogenetic analyses was inferred using maximum likelihood (TN+G+I) and Bayesian inference. Numbers at nodes indicate BS/PP values. Clades that are monophyletic at the order level are shaded and indicated with a continuous line.


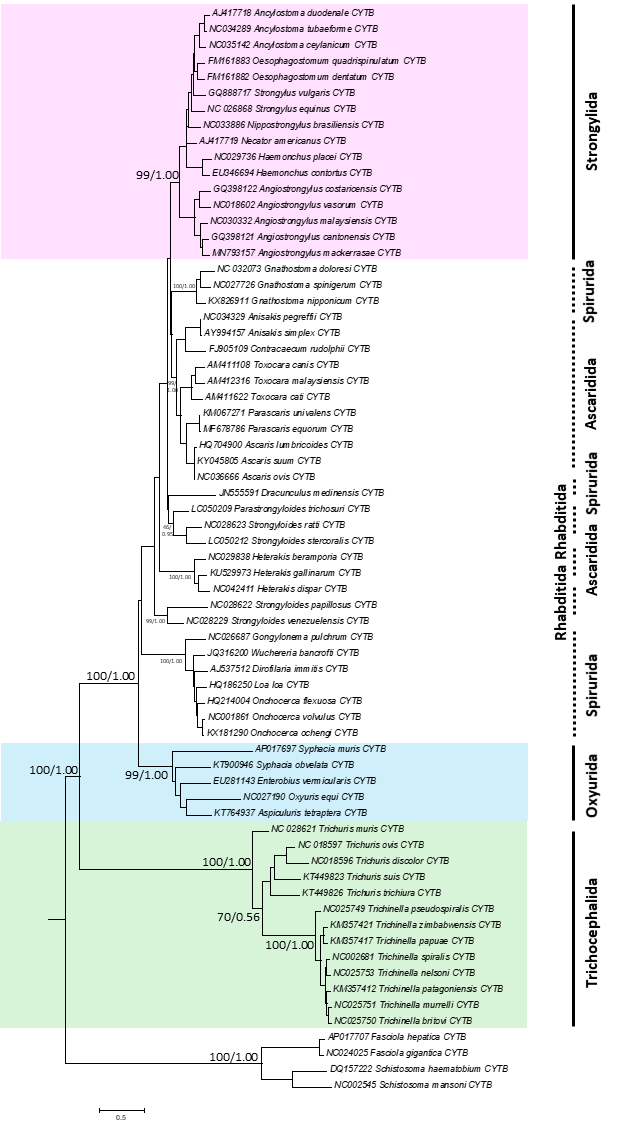


**Fig S1e. Phylogeny using *cytB* gene sequences as genetic marker for nematodes.**

Phylogenetic analyses was inferred using maximum likelihood (GTR+G+I) and Bayesian inference. Numbers at nodes indicate BS/PP values. Clades that are monophyletic at the order level are shaded and indicated with a continuous line.


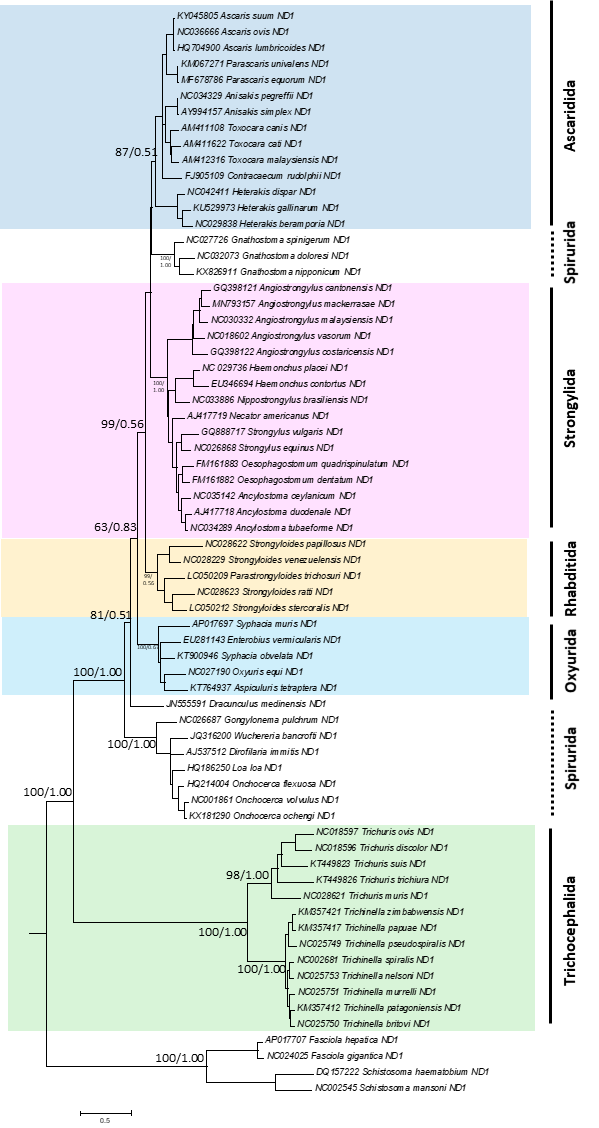


**Fig S1f. Phylogeny using *NAD1* gene sequences as genetic marker for nematodes.**

Phylogenetic analyses was inferred using maximum likelihood (TN) and Bayesian inference. Numbers at nodes indicate BS/PP values. Clades that are monophyletic at the order level are shaded and indicated with a continuous line.


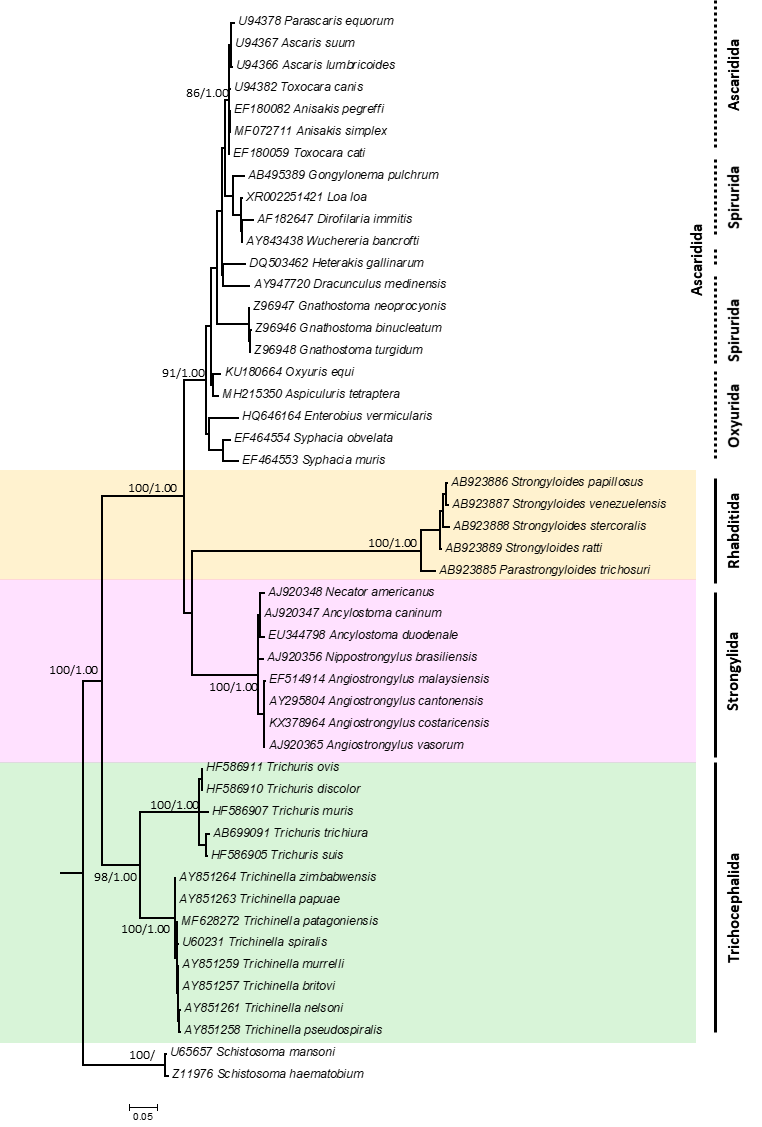


**Fig S1g. Phylogeny using 18S rRNA gene sequences as genetic marker for nematodes.**

Phylogenetic analyses was inferred using maximum likelihood (T3+G) and Bayesian inference. Numbers at nodes indicate BS/PP values. Clades that are monophyletic at the order level are shaded and indicated with a continuous line.


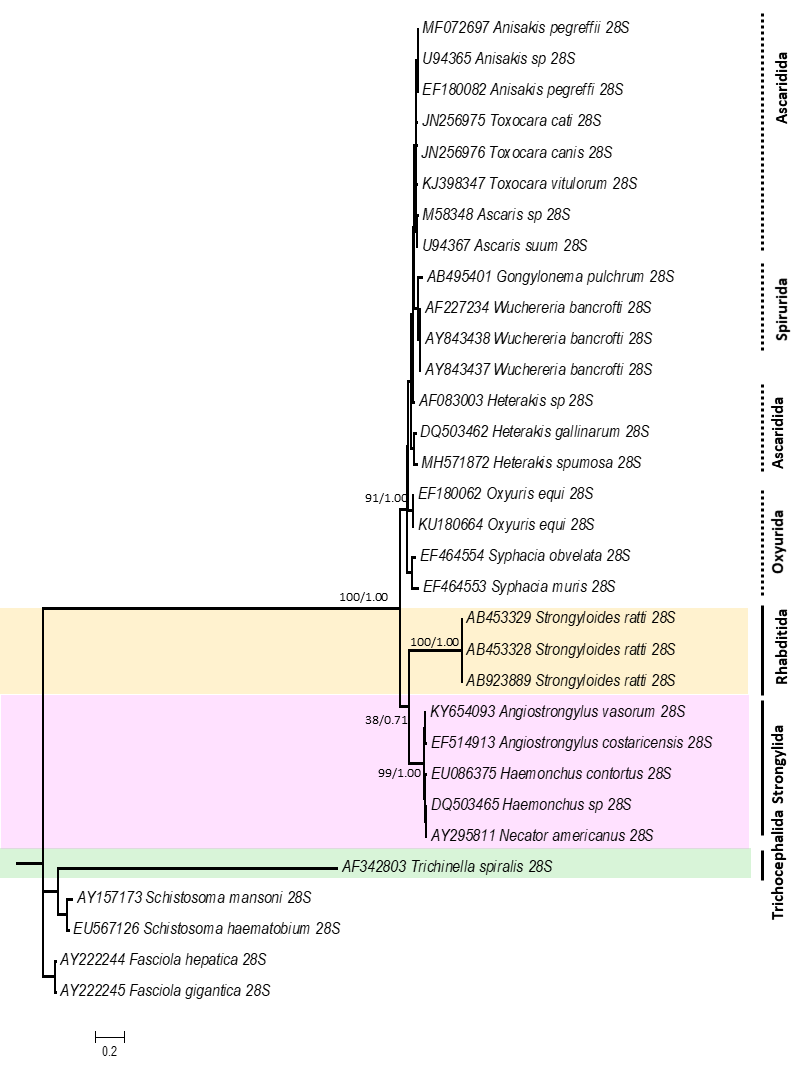


**Fig S1h. Phylogeny using 28S rRNA gene sequences as genetic marker for nematodes.**

Phylogenetic analyses was inferred using maximum likelihood (TN+G) and Bayesian inference. Numbers at nodes indicate BS/PP values. Clades that are monophyletic at the order level are shaded and indicated with a continuous line.


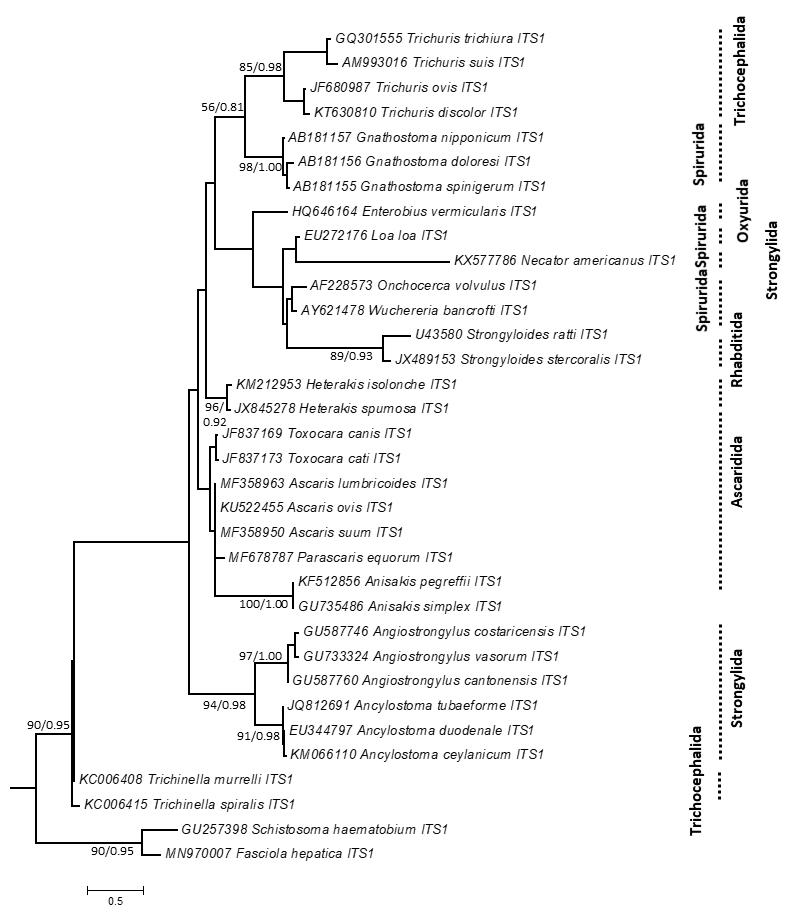


**Fig S1i. Phylogeny using ITS1 sequences as genetic marker for nematodes.**

Phylogenetic analyses was inferred using maximum likelihood (T3) and Bayesian inference. Numbers at nodes indicate BS/PP values. Clades that are monophyletic at the order level are shaded and indicated with a continuous line.


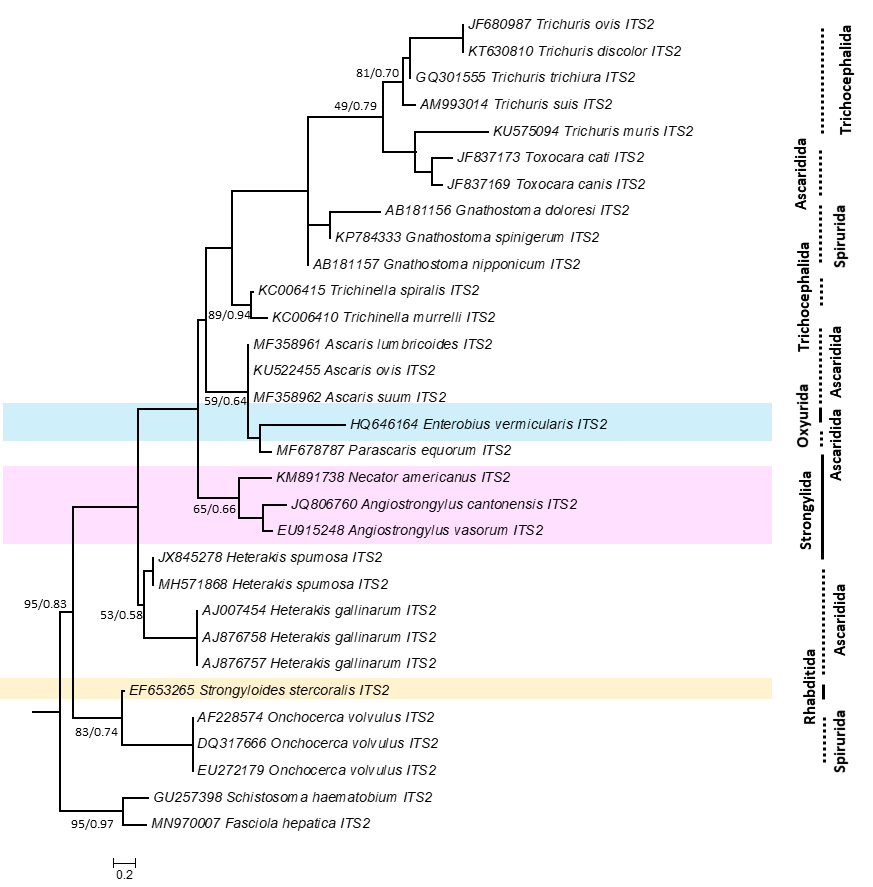


**Fig S1j. Phylogeny using ITS2 sequences as genetic marker for nematodes.**

Phylogenetic analyses was inferred using maximum likelihood (K2) and Bayesian inference. Numbers at nodes indicate BS/PP values. Clades that are monophyletic at the order level are shaded and indicated with a continuous line.


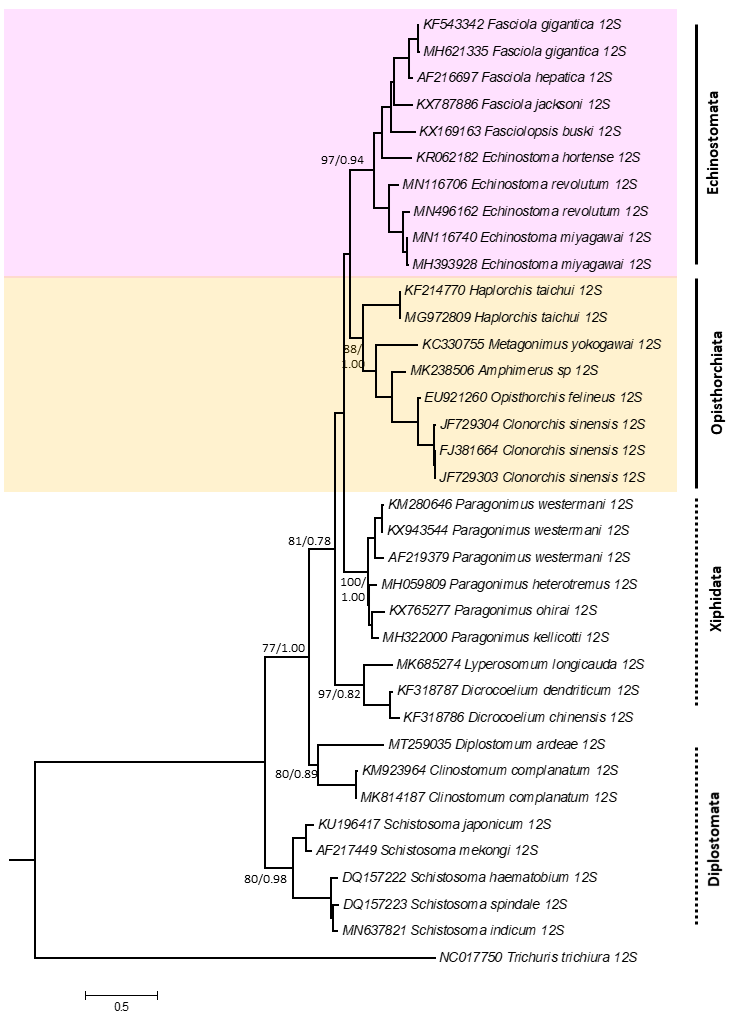


**Fig S2a. Phylogeny using 12S rRNA gene sequences as genetic marker for trematodes.**

Phylogenetic analyses was inferred using maximum likelihood (GTR+G) and Bayesian inference. Numbers at nodes indicate BS/PP values. Clades that are monophyletic at the order level are shaded and indicated with a continuous line.


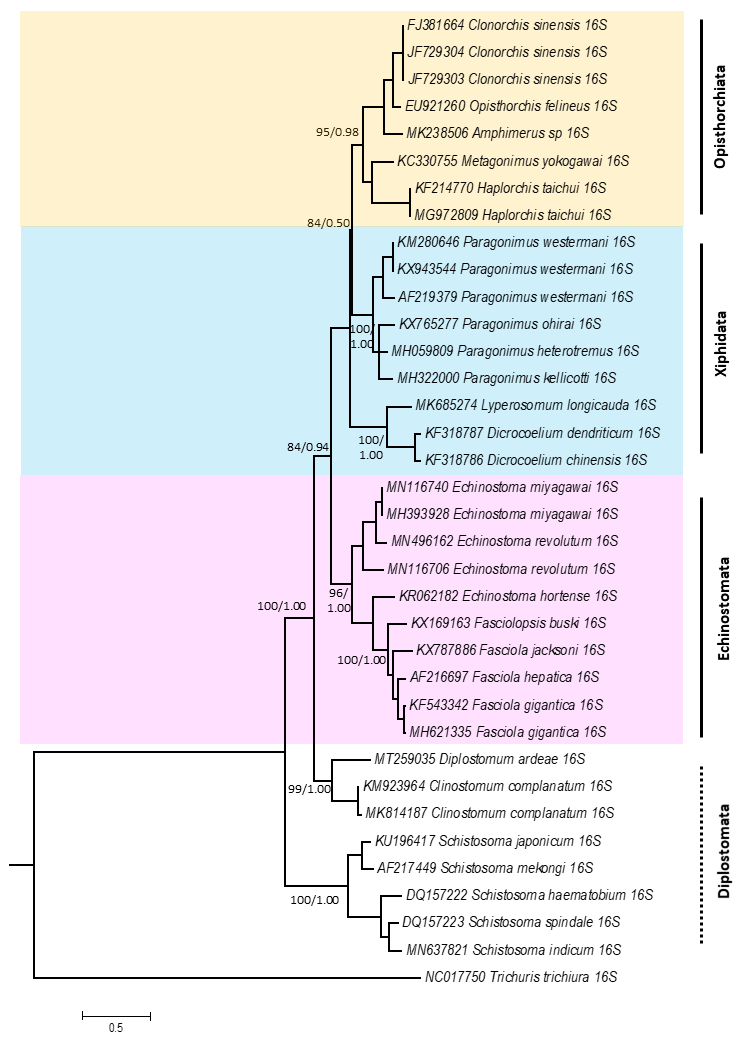


**Fig S2b. Phylogeny using 16S rRNA gene sequences as genetic marker for trematodes.**

Phylogenetic analyses was inferred using maximum likelihood (GTR+G+I) and Bayesian inference. Numbers at nodes indicate BS/PP values. Clades that are monophyletic at the order level are shaded and indicated with a continuous line.


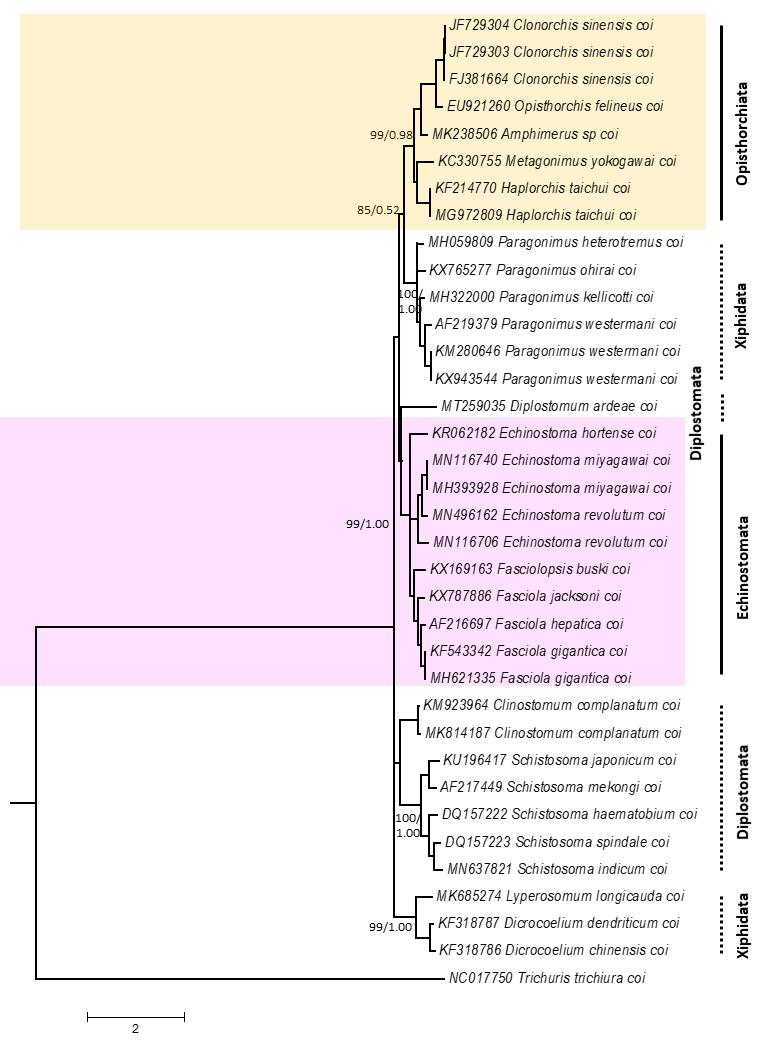


**Fig S2c. Phylogeny using *COI* sequences as genetic marker for trematodes.**

Phylogenetic analyses was inferred using maximum likelihood (GTR+G+I) and Bayesian inference. Numbers at nodes indicate BS/PP values. Clades that are monophyletic at the order level are shaded and indicated with a continuous line.


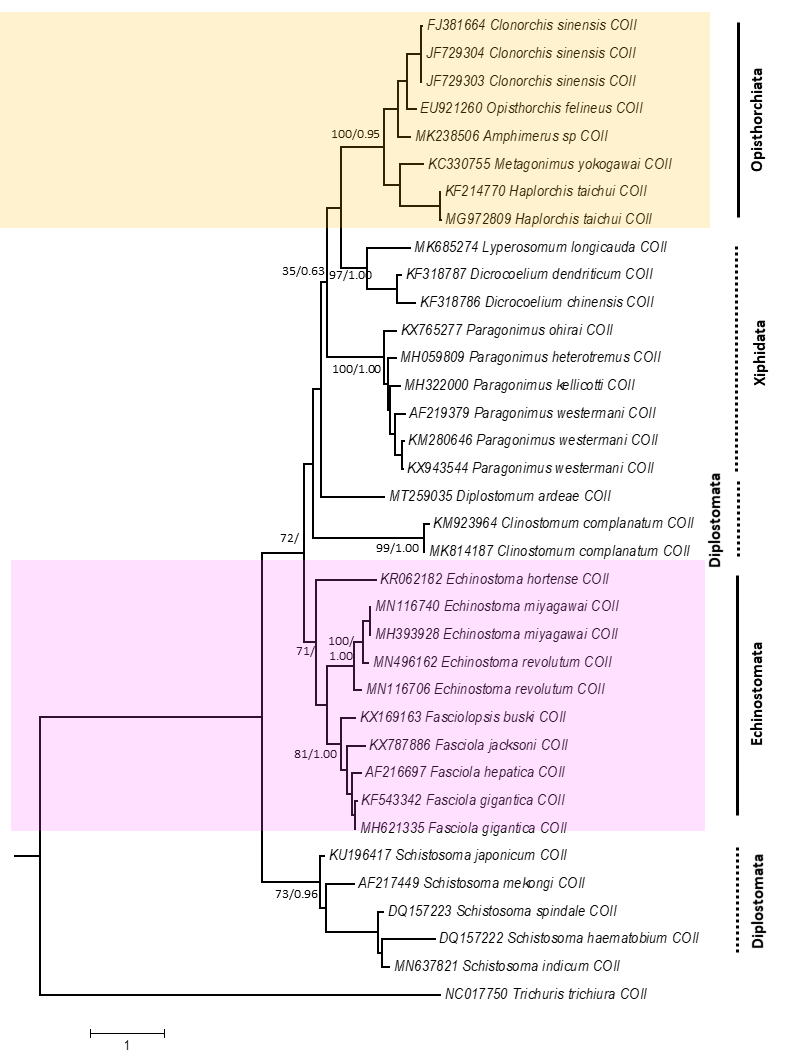


**Fig S2d. Phylogeny using *COII* sequences as genetic marker for trematodes.**

Phylogenetic analyses was inferred using maximum likelihood (GTR+G) and Bayesian inference. Numbers at nodes indicate BS/PP values. Clades that are monophyletic at the order level are shaded and indicated with a continuous line.


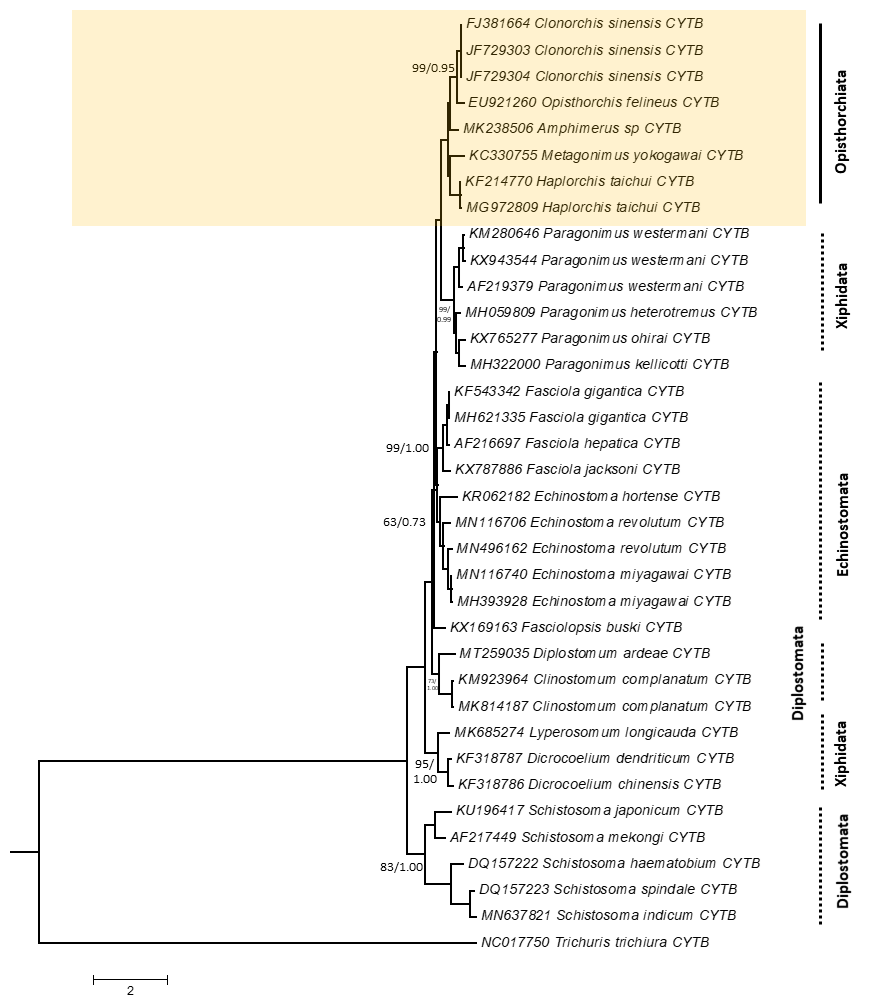


**Fig S2e. Phylogeny using *cytB* sequences as genetic marker for trematodes.**

Phylogenetic analyses was inferred using maximum likelihood (GTR+G) and Bayesian inference. Numbers at nodes indicate BS/PP values. Clades that are monophyletic at the order level are shaded and indicated with a continuous line.


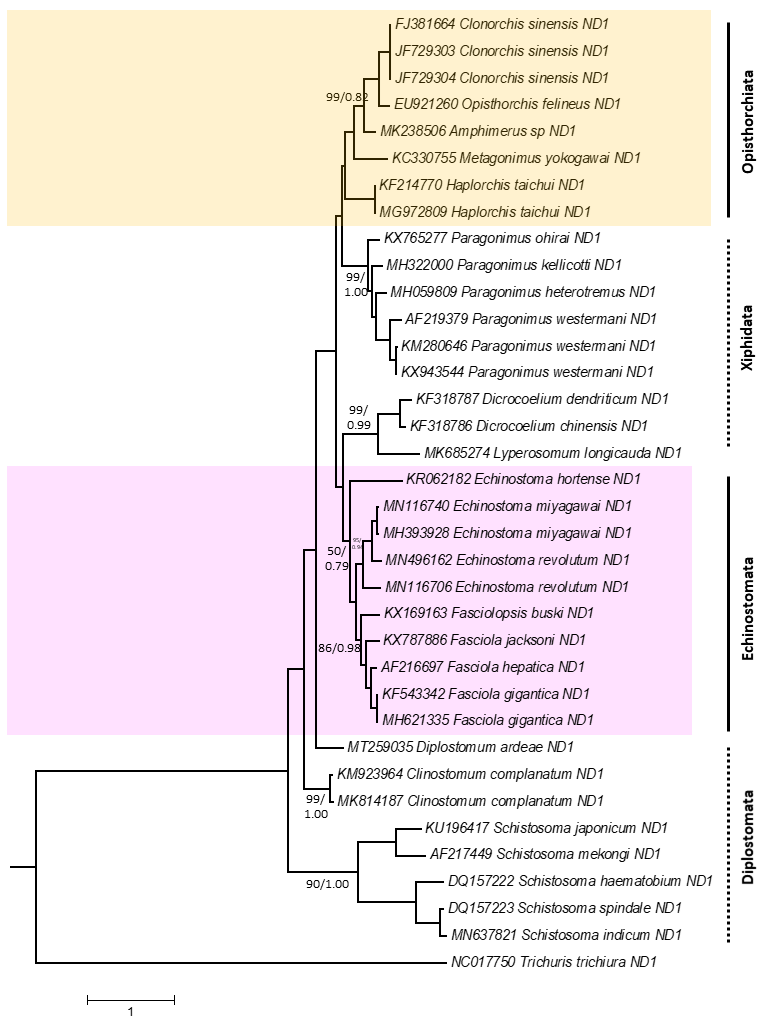


**Fig S2f. Phylogeny using *NAD1* sequences as genetic marker for trematodes.**

Phylogenetic analyses was inferred using maximum likelihood (GTR+G) and Bayesian inference. Numbers at nodes indicate BS/PP values. Clades that are monophyletic at the order level are shaded and indicated with a continuous line.


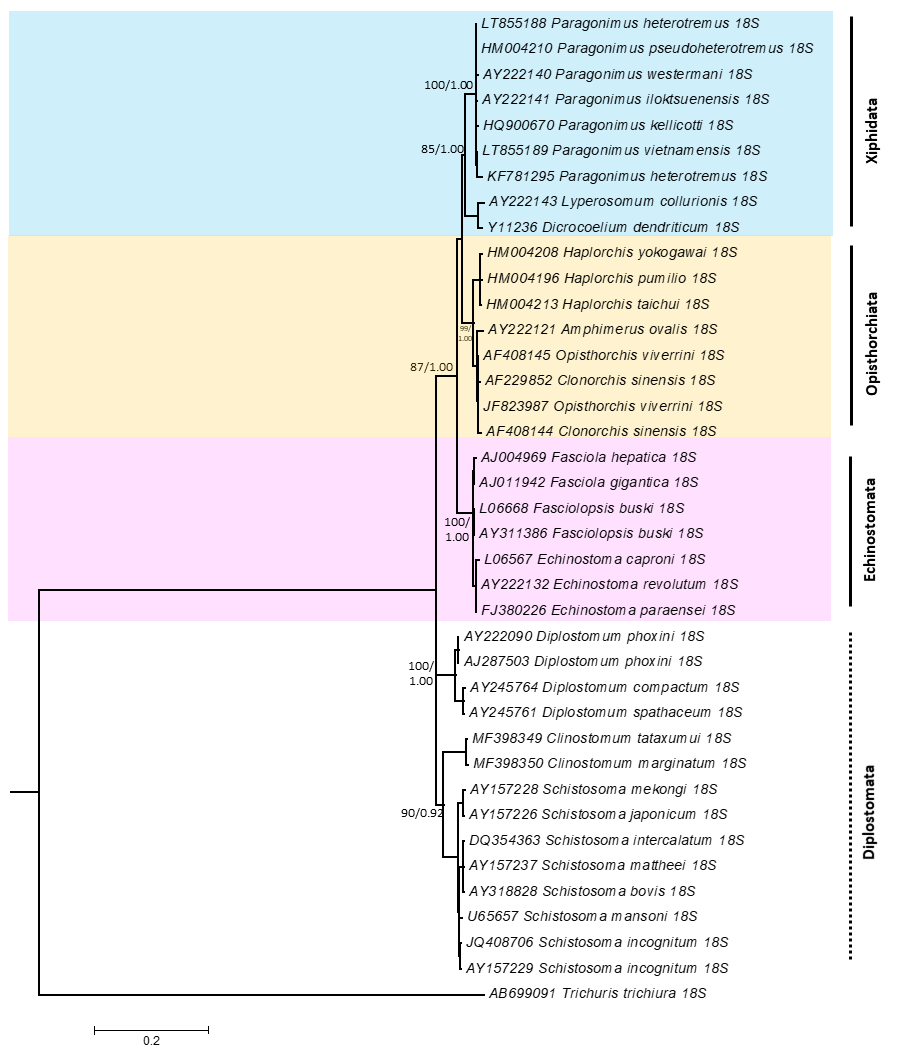


**Fig S2g. Phylogeny using 18S rRNA gene sequences as genetic marker for trematodes.**

Phylogenetic analyses was inferred using maximum likelihood (K2+G+I) and Bayesian inference. Numbers at nodes indicate BS/PP values. Clades that are monophyletic at the order level are shaded and indicated with a continuous line.


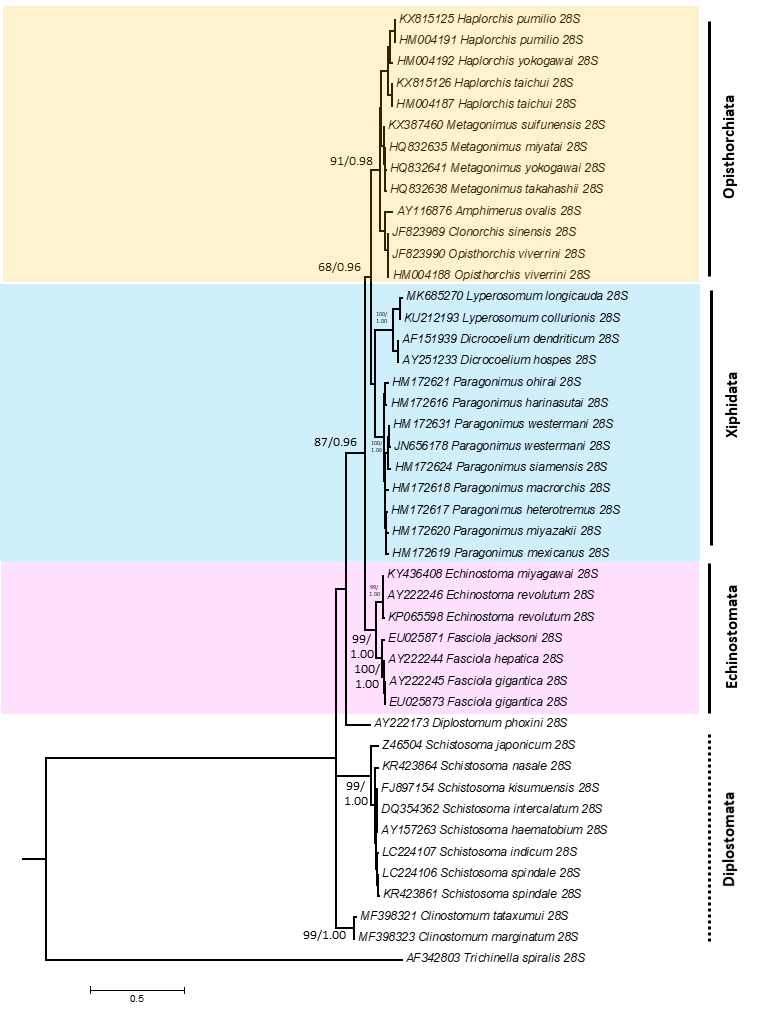


**Fig S2h. Phylogeny using 28S rRNA gene sequences as genetic marker for trematodes.**

Phylogenetic analyses was inferred using maximum likelihood (GTR+G) and Bayesian inference. Numbers at nodes indicate BS/PP values. Clades that are monophyletic at the order level are shaded and indicated with a continuous line.


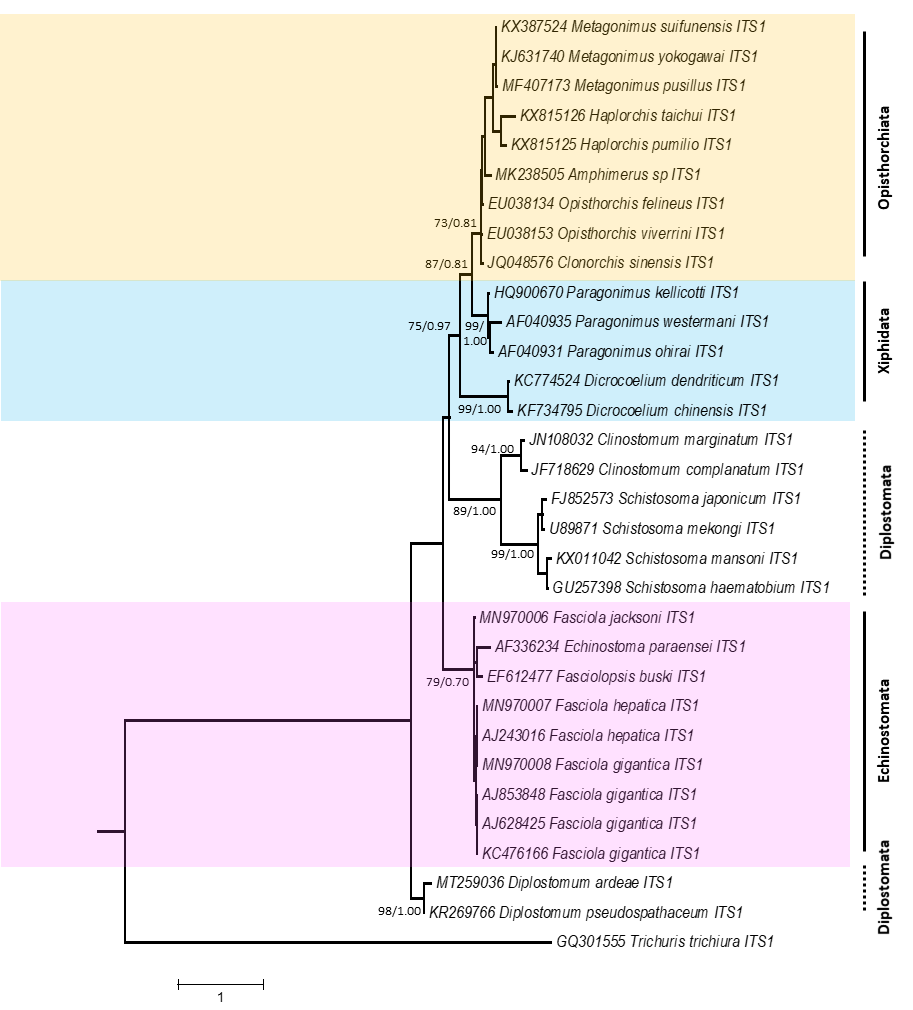


**Fig S2i. Phylogeny using ITS1 sequences as genetic marker for trematodes.**

Phylogenetic analyses was inferred using maximum likelihood (GTR+G) and Bayesian inference. Numbers at nodes indicate BS/PP values. Clades that are monophyletic at the order level are shaded and indicated with a continuous line.


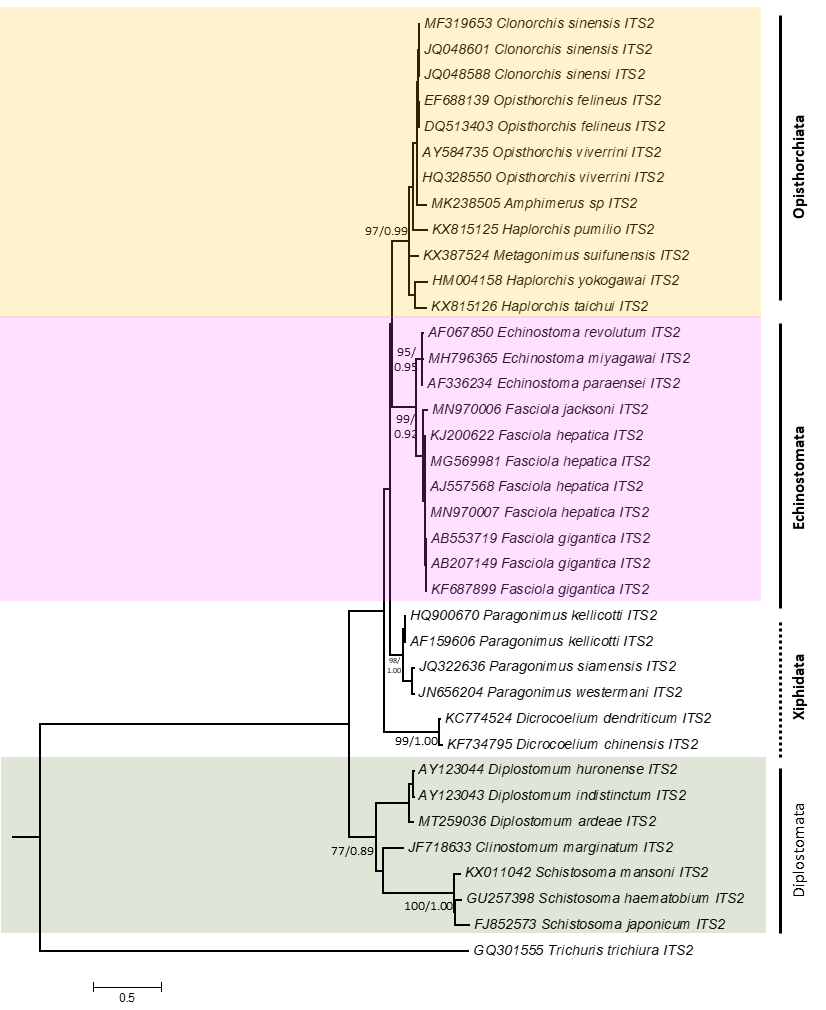


**Fig S2j. Phylogeny using ITS2 sequences as genetic marker for trematodes.**

Phylogenetic analyses was inferred using maximum likelihood (T3+G+I) and Bayesian inference. Numbers at nodes indicate BS/PP values. Clades that are monophyletic at the order level are shaded and indicated with a continuous line.


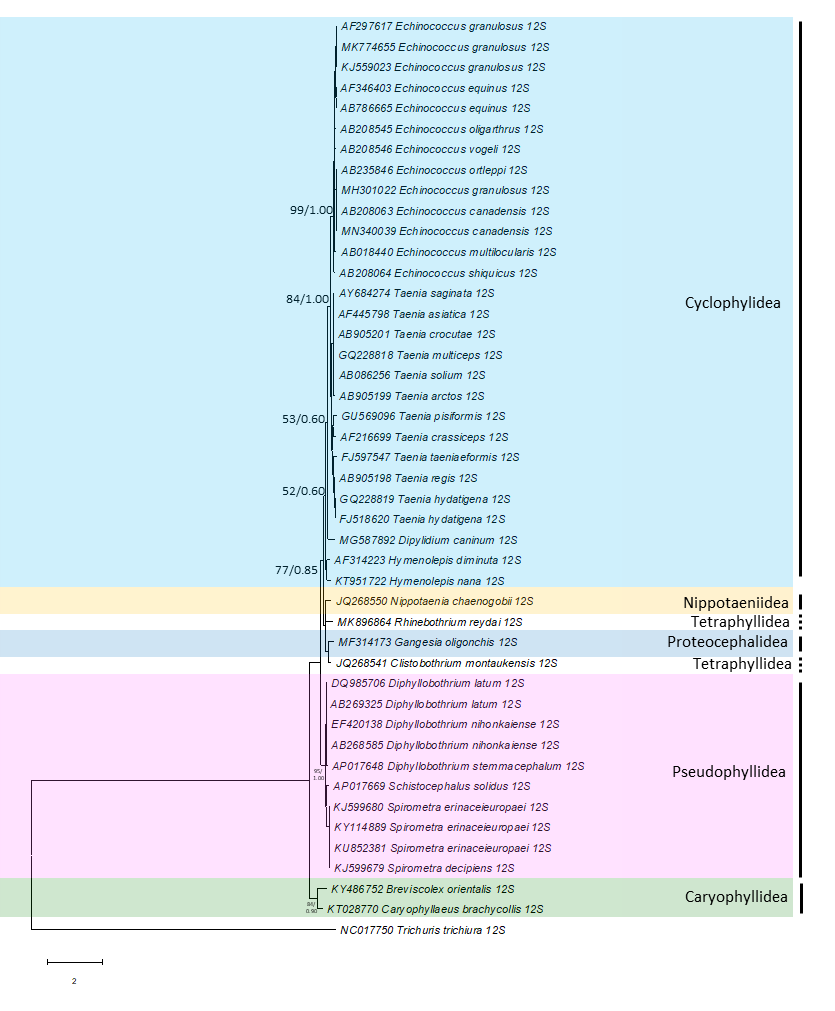


**Fig S3a. Phylogeny using 12S rRNA gene sequences as genetic marker for cestodes.**

Phylogenetic analyses was inferred using maximum likelihood (GTR+G) and Bayesian inference. Numbers at nodes indicate BS/PP values. Clades that are monophyletic at the order level are shaded and indicated with a continuous line.


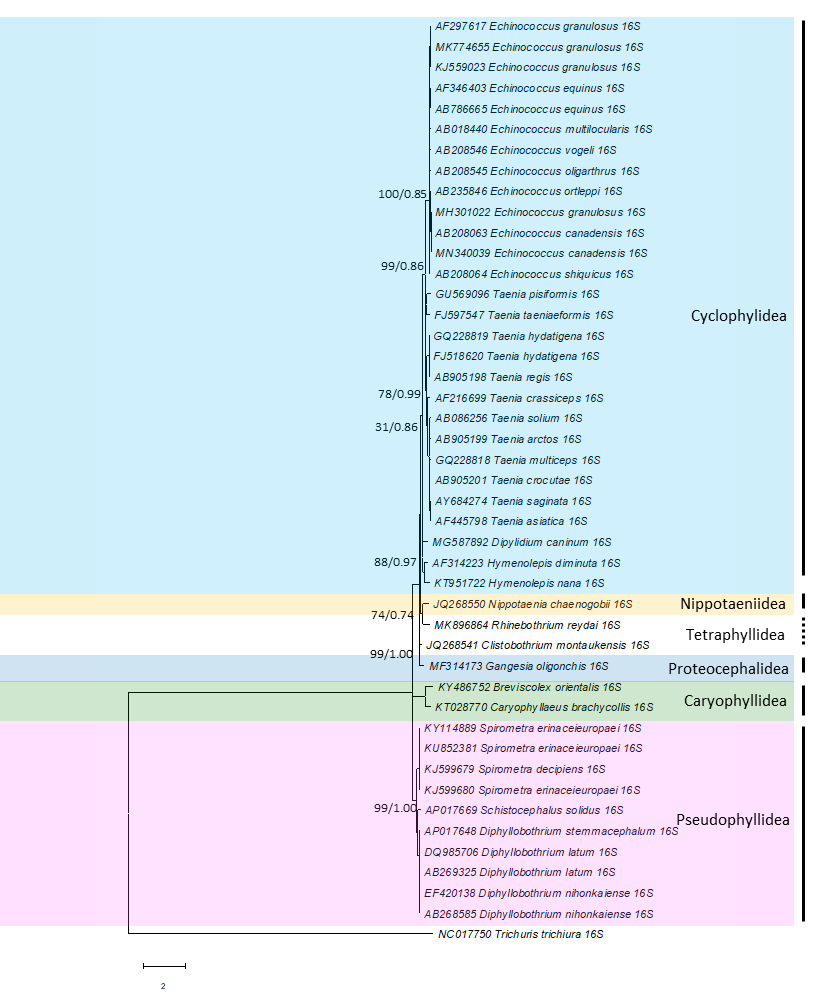


**Fig S3b. Phylogeny using 16S rRNA gene sequences as genetic marker for cestodes.**

Phylogenetic analyses was inferred using maximum likelihood (GTR+G+I) and Bayesian inference. Numbers at nodes indicate BS/PP values. Clades that are monophyletic at the order level are shaded and indicated with a continuous line.


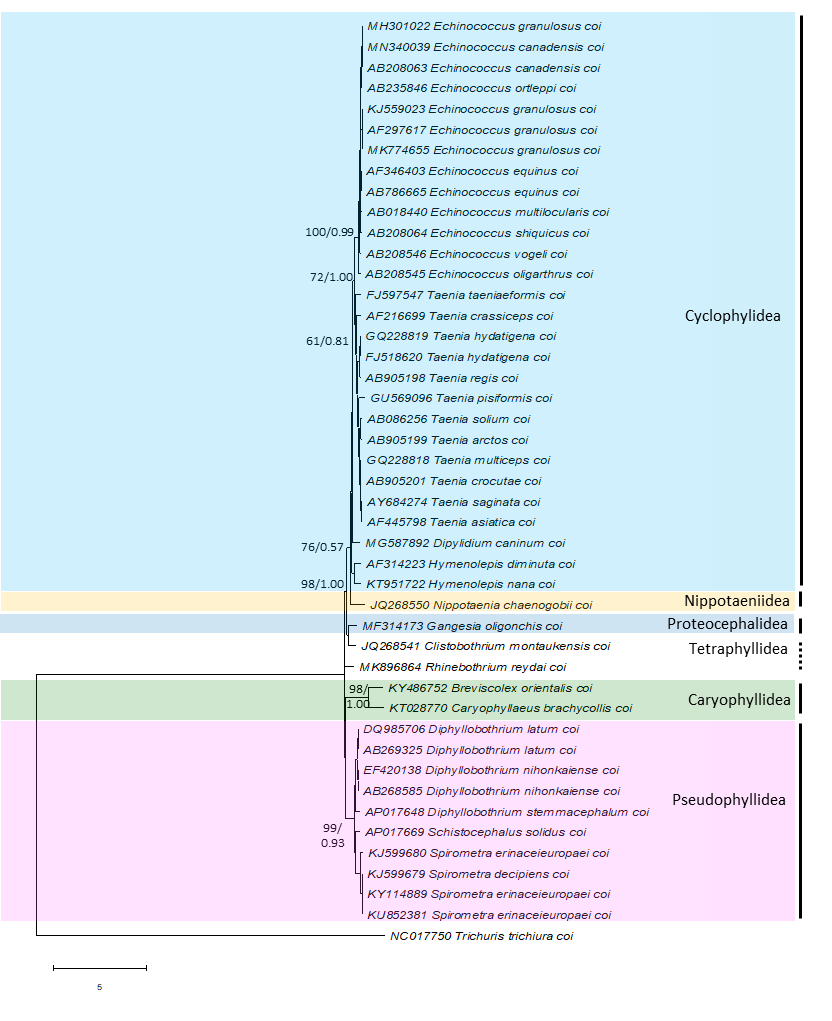


**Fig S3c. Phylogeny using *COI* gene sequences as genetic marker for cestodes.**

Phylogenetic analyses was inferred using maximum likelihood (GTR+G) and Bayesian inference. Numbers at nodes indicate BS/PP values. Clades that are monophyletic at the order level are shaded and indicated with a continuous line.


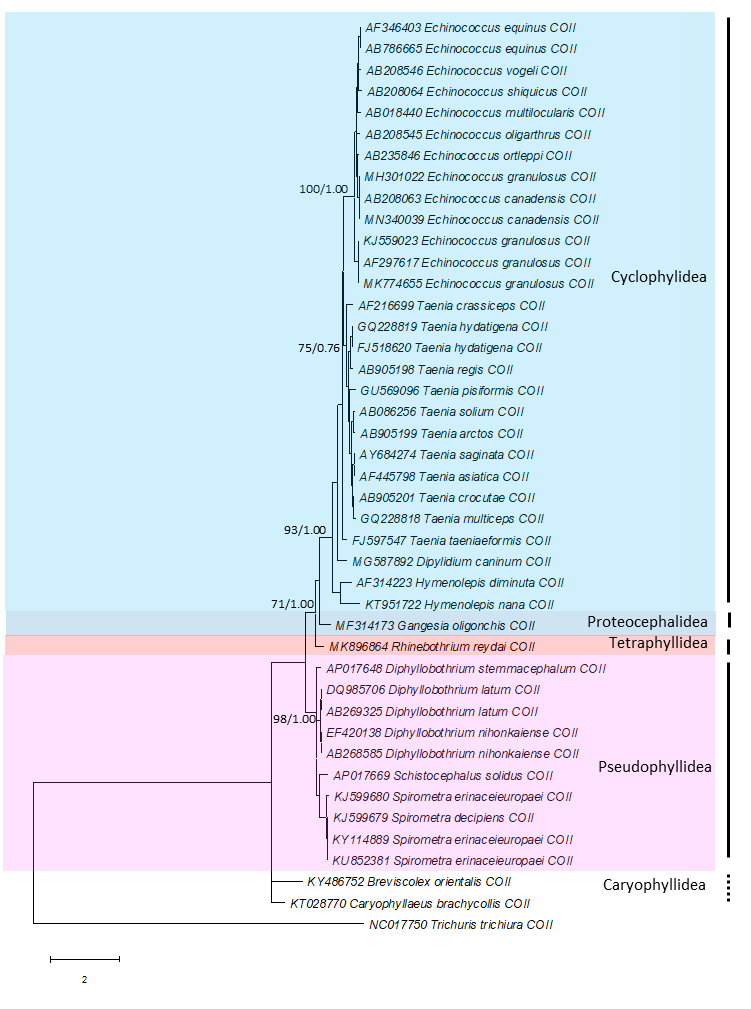


**Fig S3d. Phylogeny using *COII* gene sequences as genetic marker for cestodes.**

Phylogenetic analyses was inferred using maximum likelihood (GTR+G) and Bayesian inference. Numbers at nodes indicate BS/PP values. Clades that are monophyletic at the order level are shaded and indicated with a continuous line.


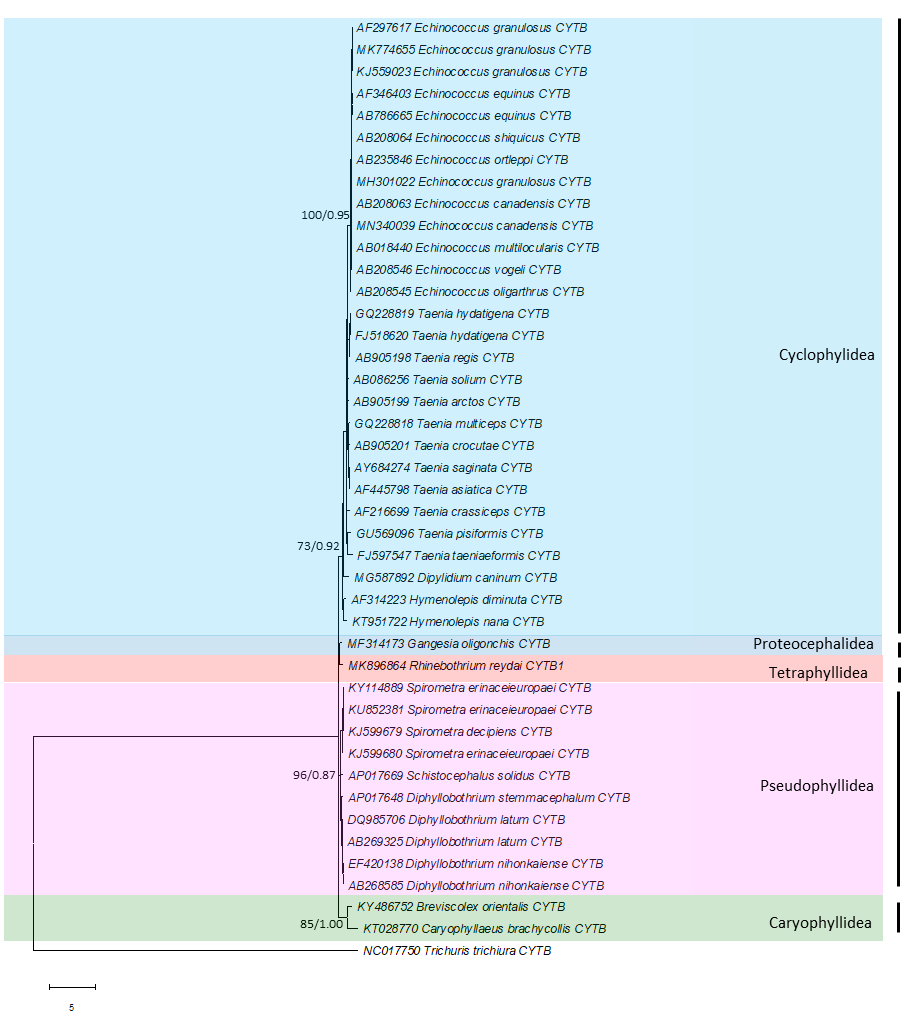


**Fig S3e. Phylogeny using *cytB* gene sequences as genetic marker for cestodes.**

Phylogenetic analyses was inferred using maximum likelihood (GTR+G+I) and Bayesian inference. Numbers at nodes indicate BS/PP values. Clades that are monophyletic at the order level are shaded and indicated with a continuous line.


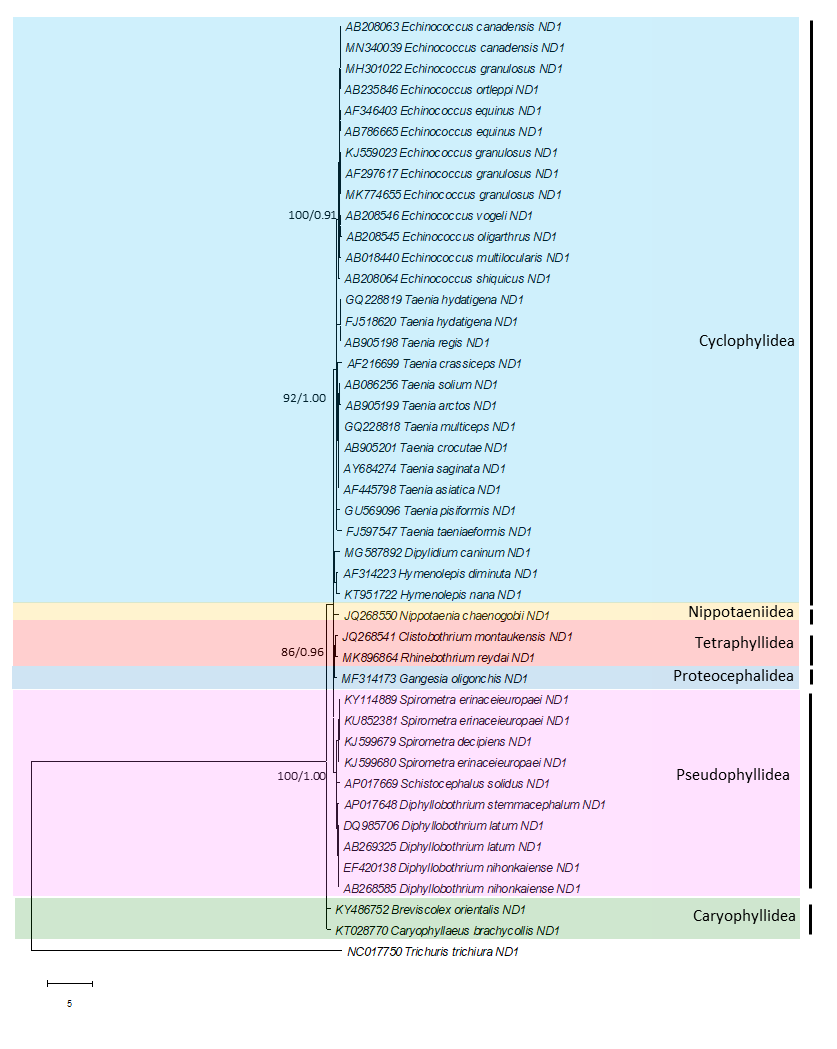


**Fig S3f. Phylogeny using *NAD1* gene sequences as genetic marker for cestodes.**

Phylogenetic analyses was inferred using maximum likelihood (GTR+G+I) and Bayesian inference. Numbers at nodes indicate BS/PP values. Clades that are monophyletic at the order level are shaded and indicated with a continuous line.


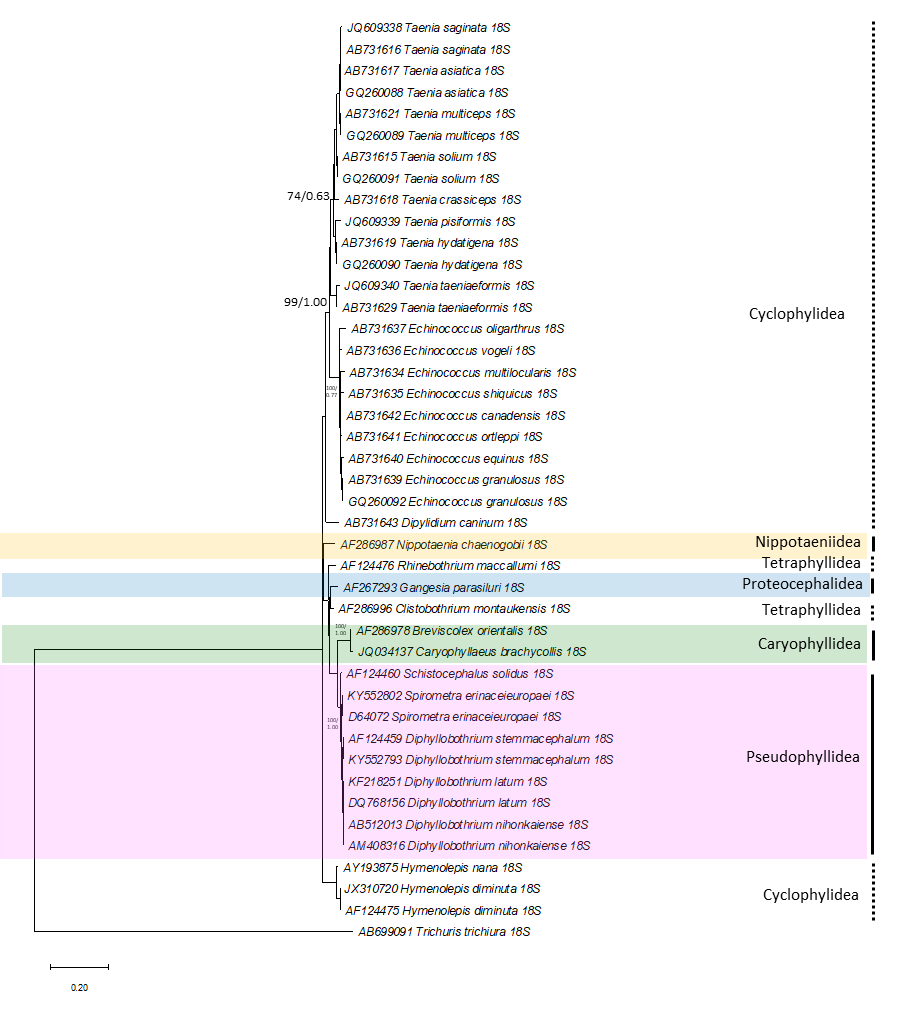


**Fig S3g. Phylogeny using 18S rRNA gene sequences as genetic marker for cestodes.**

Phylogenetic analyses was inferred using maximum likelihood (K2P+G+I) and Bayesian inference. Numbers at nodes indicate BS/PP values. Clades that are monophyletic at the order level are shaded and indicated with a continuous line.


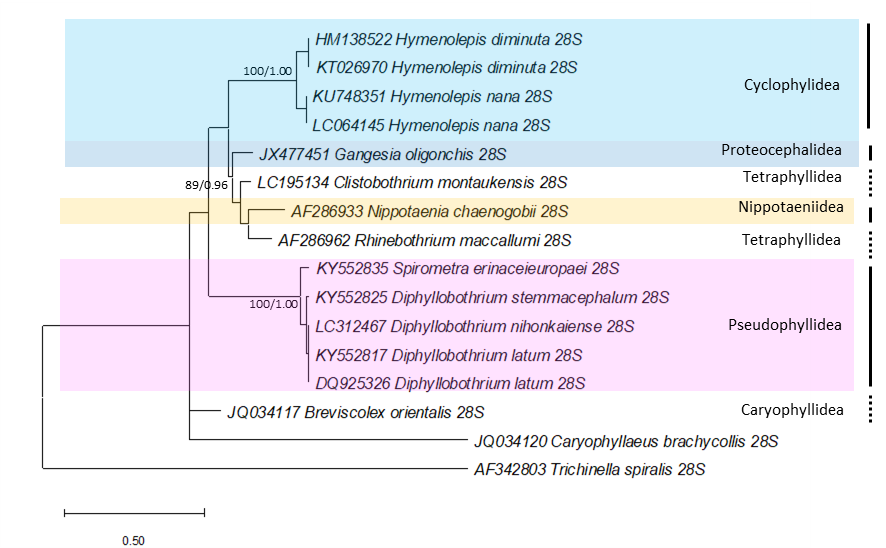


**Fig S3h. Phylogeny using 28S rRNA gene sequences as genetic marker for cestodes.**

Phylogenetic analyses was inferred using maximum likelihood (GTR+G) and Bayesian inference. Numbers at nodes indicate BS/PP values. Clades that are monophyletic at the order level are shaded and indicated with a continuous line.


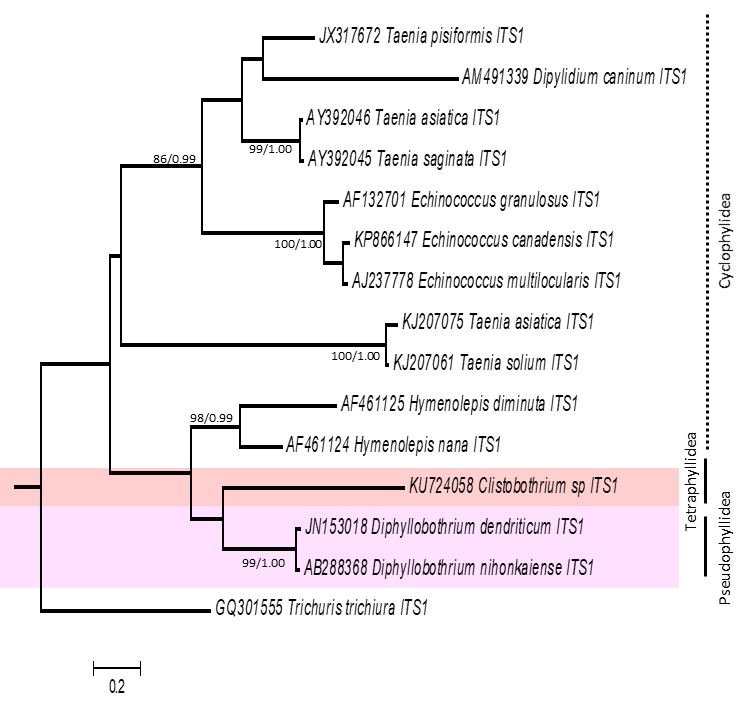


**Fig S3i. Phylogeny using ITS1 sequences as genetic marker for cestodes.**

Phylogenetic analyses was inferred using maximum likelihood (T3) and Bayesian inference. Numbers at nodes indicate BS/PP values. Clades that are monophyletic at the order level are shaded and indicated with a continuous line.


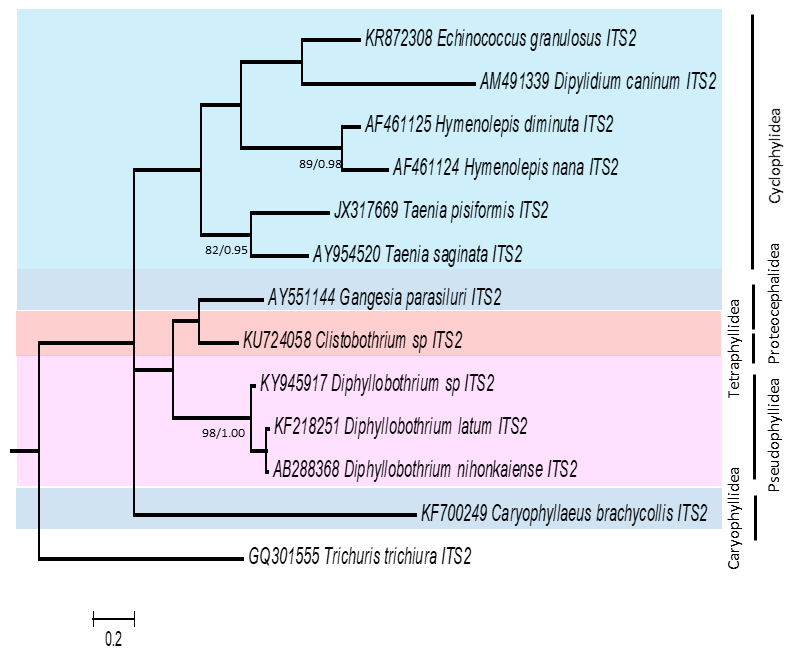


**Fig S3j. Phylogeny using ITS2 gene sequences as genetic marker for cestodes.**

Phylogenetic analyses was inferred using maximum likelihood (HKY) and Bayesian inference. Numbers at nodes indicate BS/PP values. Clades that are monophyletic at the order level are shaded and indicated with a continuous line.
