# Supplementary material for: Assessing the suitability of mitochondrial and nuclear DNA genetic markers for molecular systematics and species identification of helminths
Source: Parasit Vectors. 2021 May 1;14:233. doi: 10.1186/s13071-021-04737-y (PMC8088577; doi:10.1186/s13071-021-04737-y)
Supplement: Supplementary file 4 — Additional file 4: Table S12. General guide of the utility and limitation of each class of genetic marker for helminthes. [file 13071_2021_4737_MOESM4_ESM.docx]

**Additional file 4: Table S12.**

**General guide of the utility and limitation of each class of genetic marker for helminth**

| **Genetic marker** | **Molecular systematics** | | **Molecular identification** | |
| --- | --- | --- | --- | --- |
|  | **Utility** | **Limitation** | **Utility** | **Limitation** |
| **Nuclear rRNA** | - Not saturated - Highly conserved sequences, easy primer design | - Higher proportion of indels - Lack of full-length sequences | - Universal primers | - Insufficient variation between species - Longer amplicon size |
| **Nuclear spacer** | NA | - Saturated - Presence of repetitive sequences and different lengths - High sequence variability, difficult to design primers for broad taxa range - Lack of full-length sequences | - Sufficient sequence variation between species - Short amplicon size | - High variability can hinder universal primer design to cover broad taxa - Lack of reference sequences |
| **Mt protein-coding** | - Availability of full-length sequences - Lack of indels - Not saturated | - Higher sequence variation, difficult to design primers for broad taxa range | - Sufficient sequence variation between species - Availability of reference sequences | - High variability can hinder universal primer design to cover broad taxa - Possible presence of NUMTs |
| **Mt rRNA** | - Availability of full-length sequences - Lack of indels - Not saturated - Lesser sequence variation than Mt protein-coding genes | NA | - Sufficient sequence variation between species - Availability of reference sequences - Lesser sequence variation than Mt protein-coding genes, can design primers for broad taxa range | - Possible presence of NUMTs |
