# Supplementary material for: Assessing the suitability of mitochondrial and nuclear DNA genetic markers for molecular systematics and species identification of helminths
Source: Parasit Vectors. 2021 May 1;14:233. doi: 10.1186/s13071-021-04737-y (PMC8088577; doi:10.1186/s13071-021-04737-y)
Supplement: Supplementary file 5 — Additional file 5: Figure S4. Violin-plot of genetic distances for a nematodes b trematodes, c cestodes between family. [file 13071_2021_4737_MOESM5_ESM.docx]

**Additional file 5: Fig S4.**

**
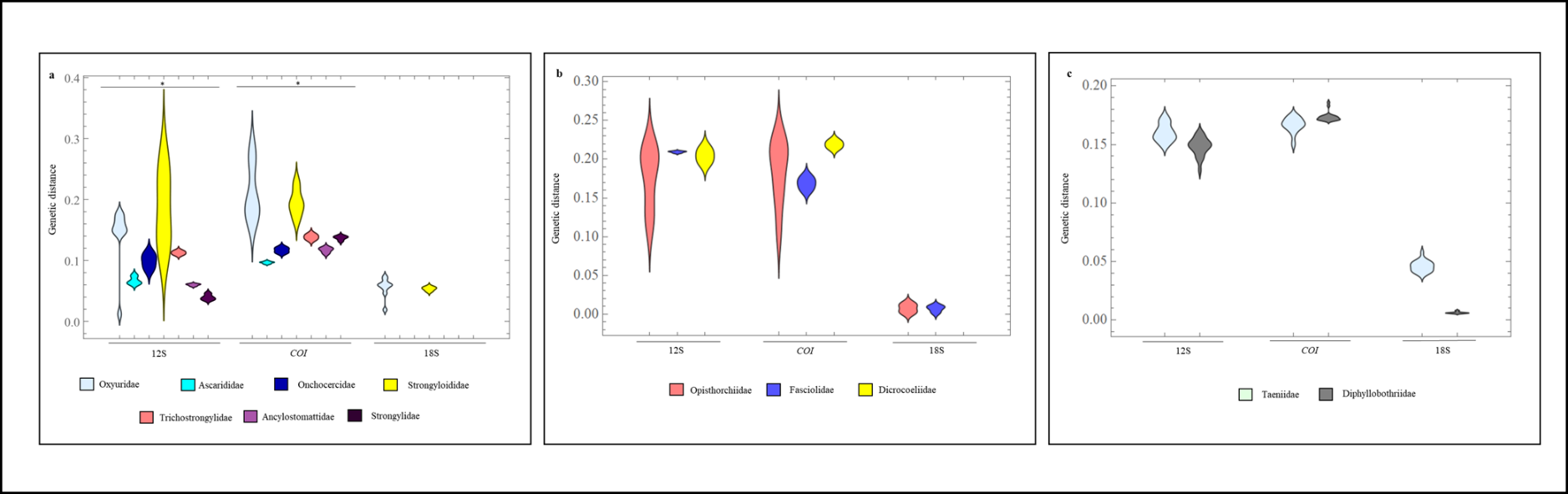
**

**Figure S4:** Violin-plot of genetic distances for **a** nematodes **b** trematodes **c** cestodes between family

*indicates statistically significant difference between each group (p < 0.001), performed using Kruskal Wallis test with Dunn’s posthoc analysis.
