# Supplementary material for: Assessing the suitability of mitochondrial and nuclear DNA genetic markers for molecular systematics and species identification of helminths
Source: Parasit Vectors. 2021 May 1;14:233. doi: 10.1186/s13071-021-04737-y (PMC8088577; doi:10.1186/s13071-021-04737-y)
Supplement: Supplementary file 7 — Additional file 7: Figures S5-S7. Plot of the estimated cut-off of each genetic marker per taxonomic level using the ‘K-means’ algorithm. [file 13071_2021_4737_MOESM7_ESM.docx]

**Additional file 7: Figs. S5 to S7.** Plot of the estimated cut-off of each genetic marker per taxonomic level using the ‘K-means’ algorithm

**
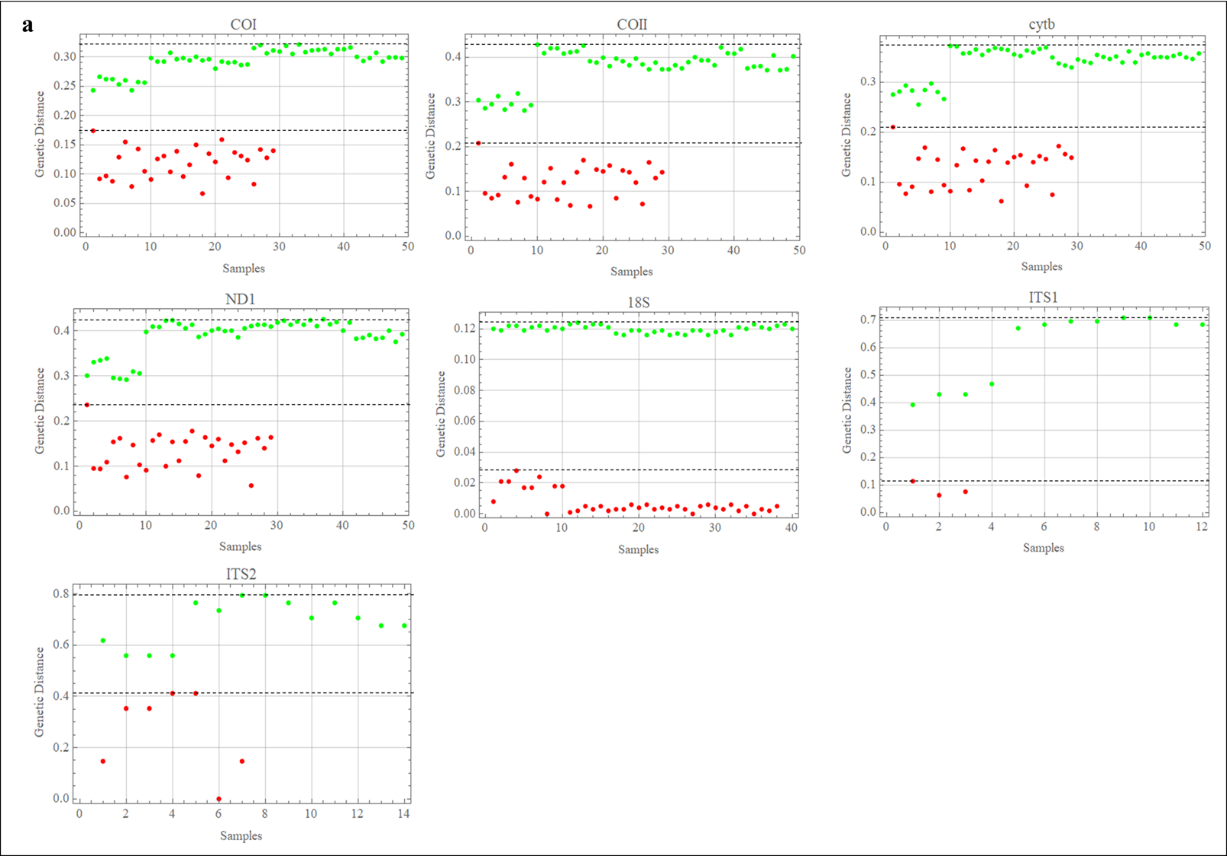
**

**
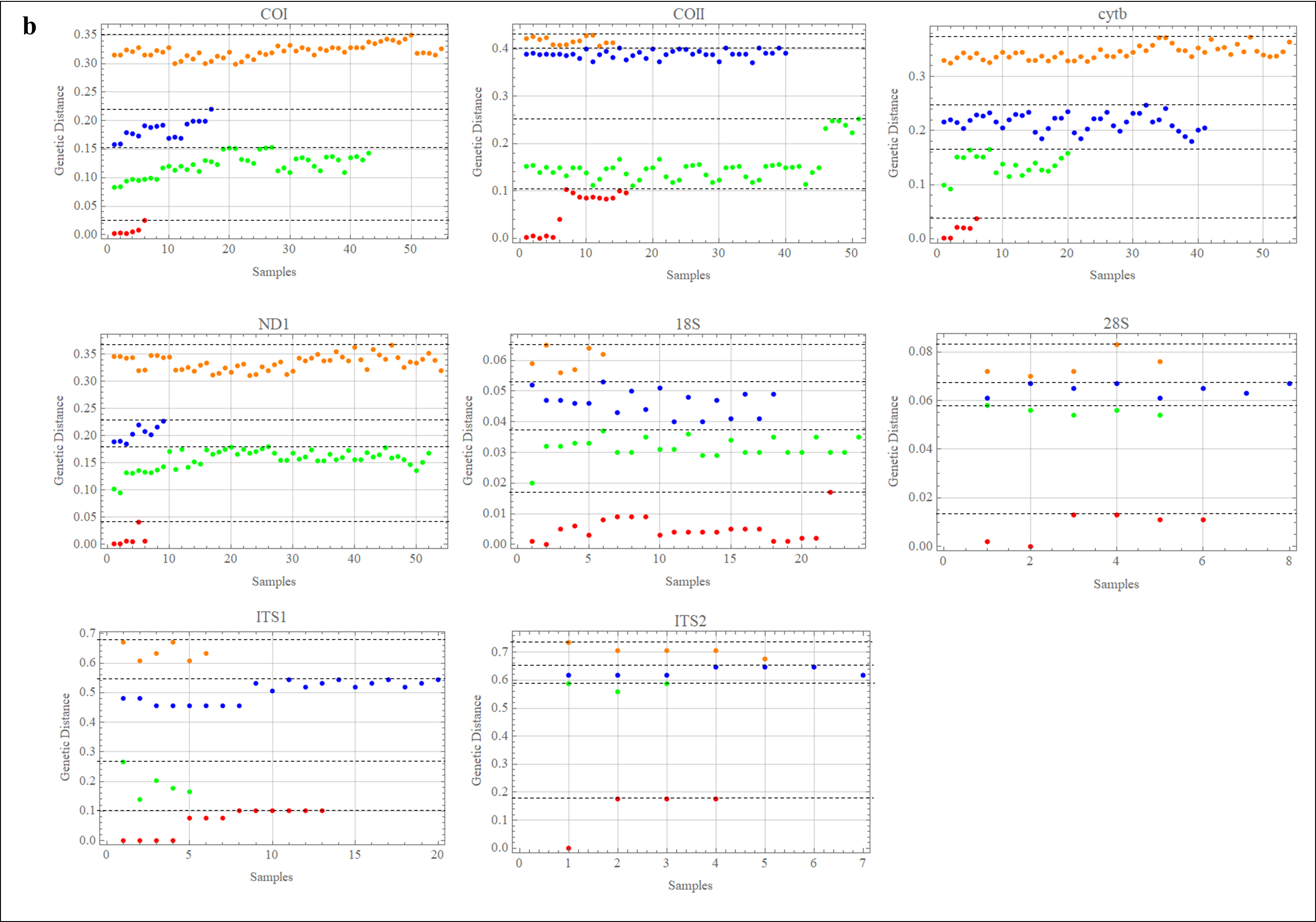
**

**
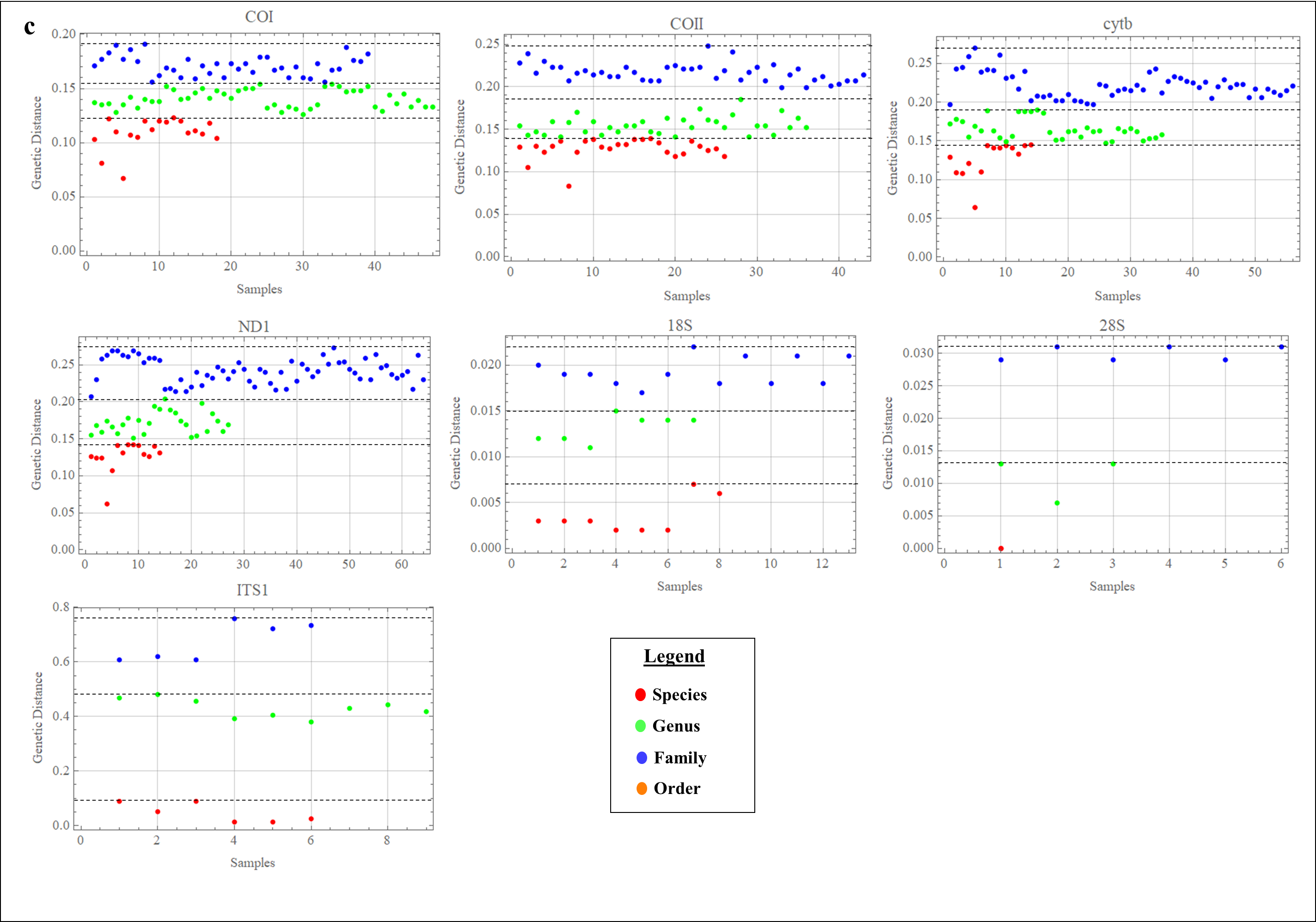
**

**Figure S5:** Plot of the estimated cut-off of each genetic marker per taxonomic level for nematodes belonging to **a** Trichocephalida **b** Ascaridida and Spirurida, **c** Strongylida using the ‘K-Means’ algorithm

Each coloured circle indicates a genetic distance value that was input into the ‘K-means’ algorithm, and the dashed lines indicate the maximum genetic distance for each taxonomic level that estimated with ‘K-means’.

**
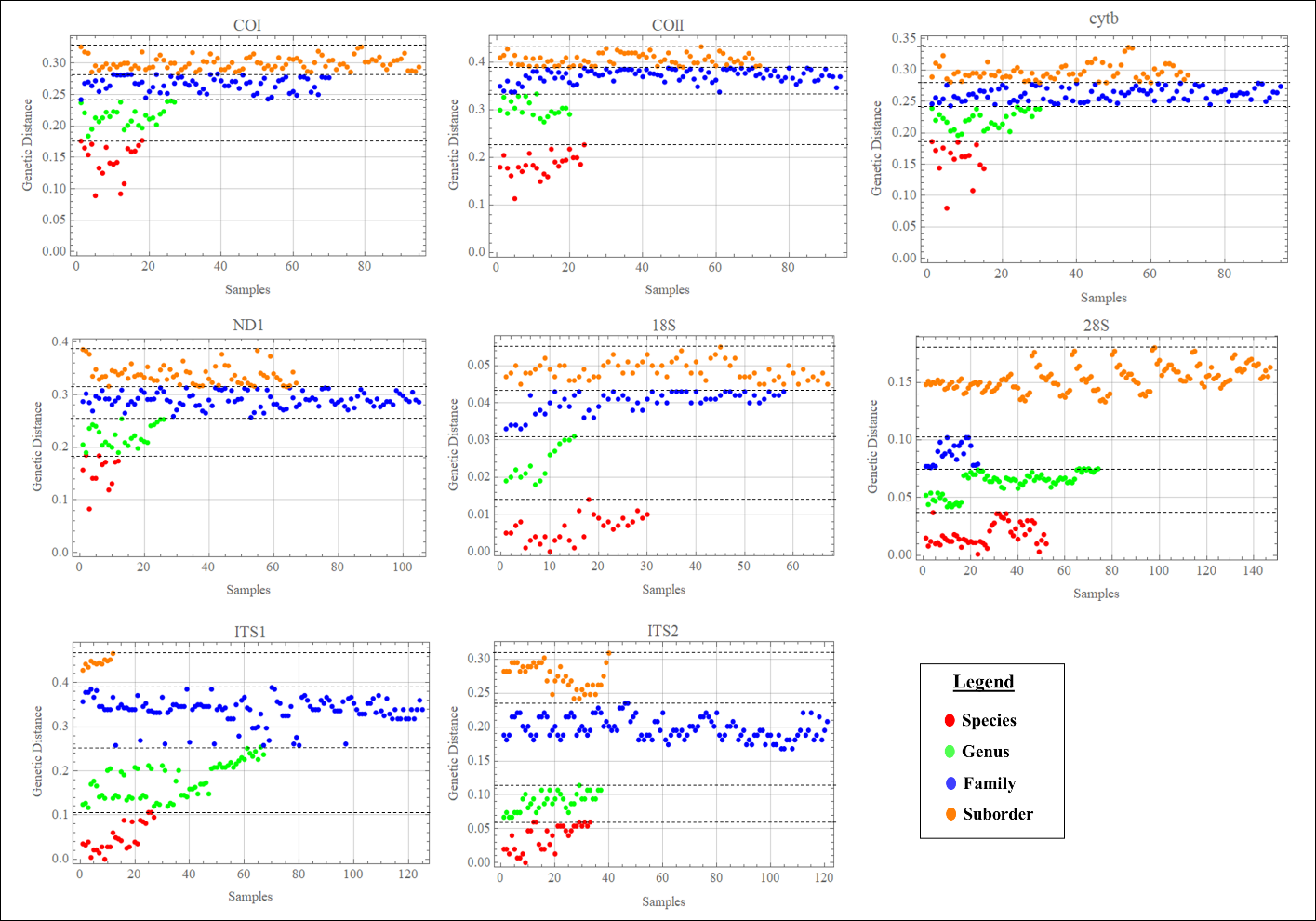
Figure S6:** Plot of the estimated cut-off of each genetic marker per taxonomic level for trematodes using the ‘K-Means’ algorithm

Each coloured circle indicate a genetic distance value that was input into the ‘K-means’ algorithm, and the dashed lines indicate the maximum genetic distance for each taxonomic level that estimated with ‘K-means’.

**
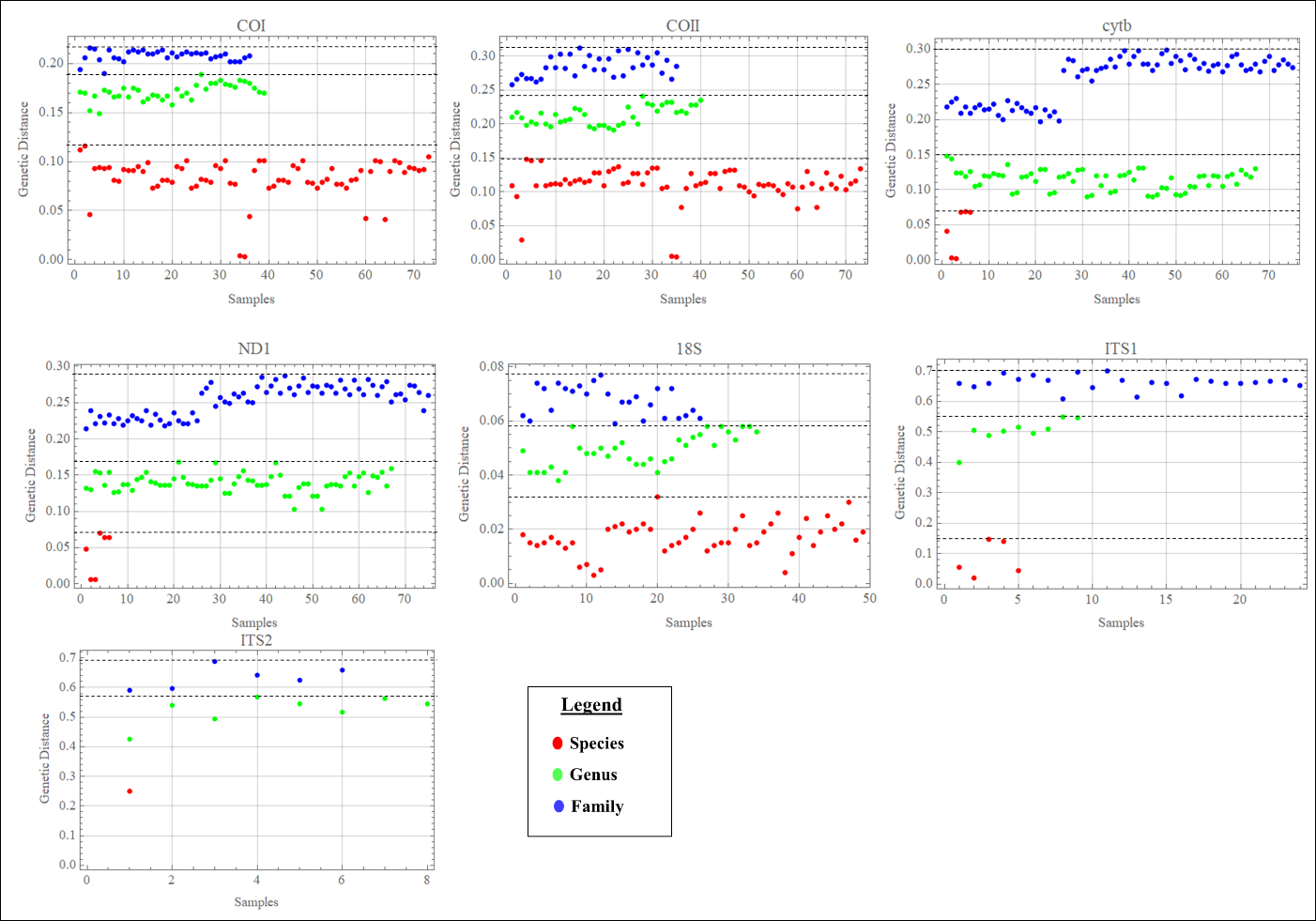
**

**Figure S7:** Plot of the estimated cut-off of each genetic marker per taxonomic level for cestodes using the ‘K-Means’ algorithm

Each coloured circle indicate a genetic distance value that was input into the ‘K-means’ algorithm, and the dashed lines indicate the maximum genetic distance for each taxonomic level that estimated with ‘K-means’.
